# Chemosensory signal transduction in *Caenorhabditis elegans*

**DOI:** 10.1093/genetics/iyab004

**Published:** 2021-03-09

**Authors:** Denise M Ferkey, Piali Sengupta, Noelle D L’Etoile

**Affiliations:** 1 Department of Biological Sciences, University at Buffalo, The State University of New York, Buffalo, NY 14260, USA; 2 Department of Biology, Brandeis University, Waltham, MA 02454, USA; 3 Department of Cell and Tissue Biology, University of California, San Francisco, CA 94143, USA

**Keywords:** WormBook, chemosensation, signal transduction, taste, odorant, pheromone, *C. elegans*, olfaction, GPCR, sensory, signaling, gustation

## Abstract

Chemosensory neurons translate perception of external chemical cues, including odorants, tastants, and pheromones, into information that drives attraction or avoidance motor programs. In the laboratory, robust behavioral assays, coupled with powerful genetic, molecular and optical tools, have made *Caenorhabditis elegans* an ideal experimental system in which to dissect the contributions of individual genes and neurons to ethologically relevant chemosensory behaviors. Here, we review current knowledge of the neurons, signal transduction molecules and regulatory mechanisms that underlie the response of *C. elegans* to chemicals, including pheromones. The majority of identified molecules and pathways share remarkable homology with sensory mechanisms in other organisms. With the development of new tools and technologies, we anticipate that continued study of chemosensory signal transduction and processing in *C. elegans* will yield additional new insights into the mechanisms by which this animal is able to detect and discriminate among thousands of chemical cues with a limited sensory neuron repertoire.

## Introduction 

The first comprehensive review of *C. elegans* chemosensation was published in WormBook in 2006 (Bargmann 2006). This review summarized our understanding of chemosensation in the nematode at that time, beginning with work initiated in the 1970s when *C. elegans* was first being developed as a laboratory model system. In the 15 years since its publication, the number of labs studying chemosensation has grown considerably, along with our understanding of *C. elegans* nervous system function.

In this study, we focus specifically on behavioral responses of *C. elegans* to attractants and repellents, chemosensory neuron physiology, and chemosensory signal transduction molecules and pathways. We also briefly discuss behavioral plasticity, but only in the context of intracellular regulation of signaling cascades. By necessity, several salient topics have been omitted, including gas sensation, neuromodulation, and the mechanisms by which chemical information is processed and relayed to other neurons within sensory circuits (*e.g.*, downstream interneurons). The ability of several *C. elegans* sensory neurons to detect multiple classes of stimuli (polymodality) also is not explicitly covered, but this ability suggests that, given a limited number of neurons, polymodality may be necessary to achieve maximum functionality. Other nonchemosensory functions of a subset of these neurons are described elsewhere (Goodman and Sengupta 2019).

## Attractants and repellents

To effectively utilize *C. elegans* as a model system to study sensory neurobiological principles, systematic screens of worm responses to individual chemicals have been conducted over the years, beginning in the 1970s (Dusenbery 1973, 1974, 1975; Ward 1973). Table 1 provides a nonexhaustive list of water-soluble and volatile compounds that have been demonstrated to attract or repel wild-type animals (defined here as the Bristol N2 strain) in the laboratory. Furthermore, *C. elegans* can discriminate between many of these compounds (Chou *et al.* 1996; L'Etoile and Bargmann 2000). As in other animals, the behavioral responses of *C. elegans* to a specific chemical can depend on its concentration. For instance, a subset of the chemical cues that are attractive at low concentrations can elicit avoidance responses at high concentrations (Table 1).

**Table 1:** A nonexhaustive list of compounds that attract or repel wild-type animals in the laboratory and the neurons demonstrated to detect them

Chemical stimulus	Neuron(s)	Soluble (S) or Volatile (V)	Reference(s)
**Attractants**			
Cyclic nucleotides cAMP cGMP	ASE (ADF, ASG, ASI)	S	(Ward 1973) (Bargmann and Horvitz 1991)
Cations Na^+^ K^+^	ASEL (ADF, ASG, ASI) ASER (ASEL)	S	(Ward 1973) (Dusenbery 1974) (Bargmann and Horvitz 1991) (Pierce-Shimomura *et al.* 2001) (Ortiz *et al.* 2009)
Anions Cl^−^	ASER (ADF, ASG, ASI)	S	(Ward 1973) (Dusenbery 1974) (Bargmann and Horvitz 1991) (Pierce-Shimomura *et al.* 2001)
Basic pH	ASEL	S	(Ward 1973) (Dusenbery 1974) (Murayama *et al.* 2013)
Amino acids Lysine Histidine Cysteine Methionine	ASE (ASG, ASI, ASK)	S	(Ward 1973) (Bargmann and Horvitz 1991) (Ortiz *et al.* 2009)
Biotin	ASE (ADF, ASG, ASI)	S	(Bargmann and Horvitz 1991)
Pyrazine	AWA	V	(Bargmann *et al.* 1993)
Diacetyl (low)	AWA	V	(Bargmann *et al.* 1993)
Diacetyl (intermediate)^a^	AWA, AWC	V	(Chou *et al.* 2001)
2,4,5-Trimethylthiazole (low)	AWA, AWC	V	(Bargmann *et al.* 1993)
Butyric acid^b^	AWA (AWC ?)	V	(Choi *et al.* 2018)
Isobutyric acid	AWA (AWC ?)	V	(Choi *et al.* 2018)
Benzyl proprionate	AWA, AWC	V	(Choi *et al.* 2018)
Benzaldehyde (low)	AWC (AWA)	V	(Bargmann *et al.* 1993) (Leinwand *et al.* 2015)
Isoamyl alcohol (low)	AWC (AWA)	V	(Bargmann *et al.* 1993)
2-Butanone	AWC^ON^	V	(Bargmann *et al.* 1993) (Wes and Bargmann 2001)
Acetone	AWC^ON^	V	(Bargmann *et al.* 1993) (Worthy *et al.* 2018)
Dimethylthiazole	AWC	V	(Bargmann *et al.* 1993) (Choi *et al.* 2018)
1-Methylpyrrole	AWC	V	(Choi *et al.* 2018)
1-Pentanol	AWC	V	(Bargmann *et al.* 1993) (Choi *et al.* 2018)
2-Cyclohexylethanol	AWC	V	(Choi *et al.* 2018)
2-Ethoxythiazole	AWC	V	(Bargmann *et al.* 1993) (Choi *et al.* 2018)
2-Isobutylthiazole	AWC (AWA ?)	V	(Bargmann *et al.* 1993) (Choi *et al.* 2018)
2-Methylpyrazine	AWC (AWA ?)	V	(Choi *et al.* 2018)
4-Chlorobenzyl mercaptan	AWC (AWA ?)	V	(Choi *et al.* 2018)
Benzyl mercaptan	AWC (AWA ?)	V	(Choi *et al.* 2018)
2-Heptanone	AWC^ON^	V	(Bargmann *et al.* 1993) (Zhang *et al.* 2016)
2,3-Pentanedione (low)	AWC^OFF^	V	(Chou *et al.* 2001) (Wes and Bargmann 2001)
2,3-Pentanedione (intermediate) ^c^	AWA, AWC	V	(Chou *et al.* 2001)
**Repellents (avoidance)**			
Acidic pH	ASH, ADF, ASK, ASE	S	(Dusenbery 1974) (Sambongi *et al.* 2000)
Basic pH (>10.5)	ASH	S	(Sassa *et al.* 2013)
Copper	ASH, ADL, ASE	S	(Bargmann *et al.* 1990)^c^ (Sambongi *et al.* 1999)
Cadmium	ASH, ADL, ASE	S	(Sambongi *et al.* 1999)
SDS	ASH (ASK, ASI, ASJ) PHA, PHB (antagonistic)	S	(Bargmann *et al.* 1990)^c^ (Hilliard *et al.* 2002) (Liu *et al.* 2018)
Bitters quinine	ASH (ASK)	S	(Hilliard *et al.* 2004)
Diacetyl (high)	ASH	V	(Yoshida *et al.* 2012) (Taniguchi *et al.* 2014)
2,4,5-Trimethylthiazole (high)		V	(Bargmann *et al.* 1993) (Yoshida *et al.* 2012)
Benzaldehyde (high)	ASH (AWB)	V	(Bargmann *et al.* 1993) (Troemel *et al.* 1995) (Luo *et al.* 2008) (Yoshida *et al.* 2012)
Isoamyl alcohol (high)	ASH (ADL, AWB)	V	(Luo *et al.* 2008) (Yoshida *et al.* 2012)
Alcohols 1-Octanol (100%) 1-Octanol (30%)	ASH (ADL, AWB—off food) ASH	V	(Bargmann *et al.* 1993) (Troemel *et al.* 1995) (Troemel *et al.* 1997) (Chao *et al.* 2004)
Ketones 2-Nonanone	AWB (ASH)	V	(Bargmann *et al.* 1993) (Troemel *et al.* 1997) (Tanimoto *et al.* 2017)
Serrawettin W2	AWB	S	(Pradel *et al.* 2007)
Phenazine-1-carboxamide	ASJ	S	(Meisel *et al.* 2014)
Pyochelin	ASJ	S	(Meisel *et al.* 2014)
Dodecanoic acid	ASH (ADL ?, ADF ?) PHA PHB	S	(Tran *et al.* 2017)

The references include those first reporting behavioral response to the chemicals, as well as those demonstrating the neurons involved in the response. The roles of most neurons were shown by cell ablation, although some were revealed via genetic mutation or calcium imaging. Neurons with a more minor role are indicated by a smaller font. Question marks indicate neurons with a possible role in detecting a stimulus.

a Chou *et al.* (2001) refers to 1:10 dilutions of diacetyl and 2,3-pentanedione as “high” concentration. We have indicated them here as “intermediate” to distinguish it from undiluted diacetyl and 2,3-pentanedione, which animals avoid (Yoshida *et al.* 2012).

b Butyric acid was previously reported to be a neutral compound (Bargmann *et al.* 1993).

c J. Thomas unpublished, cited in Bargmann *et al.* (1990).

Beyond having a catalog of the compounds that *C. elegans* can respond to, an understanding of what each compound might represent to the nematode in the wild provides context for its neuroanatomy, physiology, and sensory integration. In its natural habitat, *C. elegans* is typically associated with microbe-rich organic matter such as rotting fruit and vegetable matter (and also slugs) (Frézal and Félix 2015). A wide variety of bacterial strains have been found along with *C. elegans* in the wild, and several nonpathogenic nutritious bacterial strains (*Alcaligenes sp*. JUb4, *Providenica sp*. JUb5, *Providencia sp*. JUb39, and *Flavobacteria sp*. JUb43) release the “fruity” smelling attractive volatiles isoamyl alcohol, ethyl isobutyrate, and ethyl isovalerate (Samuel *et al.* 2016; Schulenburg and Félix 2017; Worthy *et al.* 2018a). The well-studied attractant diacetyl is also released from a *Lactobacillus* species that was found in rotting citrus (yazu) fruit that also contained *C. elegans* (Choi *et al.* 2016). While the natural prey of *C. elegans* have not been definitively identified (Schulenburg and Félix 2017), the volatile chemicals emitted by these bacteria likely provide long-range attractive cues for seeking food.

Not all soil microbes are beneficial for *C. elegans*, and there are nematocidal fungi and bacteria that exude chemical cues that *C. elegans* avoids. For example, the pathogenic bacteria *Serratia marcescens* releases the cyclic lipodepsipentapeptide compound serrawettin W2 (Pradel *et al.* 2007), the pathogen *Pseudomonas aeruginosa* emits phenazine-1-carboxamide (PCN) and the siderophore pyochelin (Meisel *et al.* 2014), and the nematocidal bacteria *Streptomyces* secretes dodecanoic acid (Tran *et al.* 2017)—all of which repel *C. elegans*. Aversive odorants such as 1-octanol and 2-nonanone may also indicate the presence of fungi or pathogenic bacteria (Kaminski *et al.* 1974; Sharpell 1985). *C. elegans* may also use chemical cues to be alerted to the presence of hungry nematode predators such as *Pristionchus pacificus* that release soluble repellent sulfolipids when they are starved (Liu *et al.* 2018b).

Interestingly, some nematocidal predators exploit innate attractive responses of *C. elegans* to specific compounds by releasing attractive chemicals. For example, at least one nematode-trapping fungus (*Arthrobotrys oligospora*) appears to lure its prey by releasing attractive volatile compounds that might mimic food and pheromone cues (Hsueh *et al.* 2017). In addition, the pathogenic bacterium *B. nematocida* B16 secretes an attractive odor bouquet that includes benzaldehyde and 2-heptanone (among others) that lures nematodes to their death via a “Trojan horse” mechanism (Niu *et al.* 2010).

Innate responses of *C. elegans* to chemicals can be modified by experience. The attractive chemicals butanone and acetone are emitted by the pathogenic bacteria *S. marcescens* and *P. aeruginosa* (Worthy *et al.* 2018b), and inexperienced worms seek out these odors. However, following pathogenic infection, animals learn to avoid these odors (Zhang *et al.* 2005). This plasticity provides a model for learning and vertical transmission of pathogenic bacterial memory (Moore *et al.* 2019). *C. elegans* is also able to associate chemicals with food or starvation and exhibit attraction or repulsion, respectively, to these conditioned chemicals (for examples, see Colbert and Bargmann 1997; Torayama *et al.* 2007; Kunitomo *et al.* 2013; Luo *et al.* 2014).

In addition to compounds produced by potentially pathogenic organisms or predators, *C. elegans* also avoids many compounds that are generally considered harmful at high concentrations (Table 1). These include heavy metals (*e.g.*, copper, and cadmium), and plant alkaloids or derivatives (*e.g.*, quinine) that are perceived as bitter by humans and are toxic for most animals (Sambongi *et al.* 1999; Hilliard *et al.* 2004). Taken together, the complex natural environment of *C. elegans* necessitates that these animals be able to sense and respond robustly and sensitively to a range of chemical cues for optimal survival and reproduction.

## Assessing behavioral and neuronal responses

Behavioral strategies underlying *C. elegans* chemotaxis have been identified by studying animal movement in controlled spatial and temporal chemical gradients (Pierce-Shimomura *et al.* 1999, 2005; Iino and Yoshida 2009; Broekmans *et al.* 2016). The behavioral strategies used by *C. elegans* to migrate toward or away from favorable (attraction) and noxious (avoidance) chemical cues, respectively, are described in the Appendix. Here, we briefly outline the most common tools and paradigms for assessing behavioral responses, and refer the reader to the Behavior methods chapter (Hart 2006) for more detailed descriptions of chemosensory assays*.* Interested readers may also wish to consult these reviews for additional relevant information: (de Bono and Maricq 2005; Bargmann 2006; Bergamasco and Bazzicalupo 2006; Sengupta 2007; Hart and Chao 2010; Lockery 2011; Hobert 2013; Walker *et al.* 2017; Metaxakis *et al.* 2018).

Population assays provide good platforms to rapidly screen for mutations that disrupt sensory function, as well as to catalog chemicals that elicit behavioral responses. Typically, population assays are performed on agar-filled Petri dishes, with a gradient emanating from a point source of a stimulus (Bargmann and Horvitz 1991a; Bargmann *et al.* 1993). Uniform concentrations of soluble and/or volatile chemicals within quadrants of a Petri dish are also used to assess preferences (Wicks *et al.* 2000; Frøkjær-Jensen *et al.* 2008; Lee *et al.* 2009). These approaches can be high throughput, and allow the assessment of responses of tens to hundreds of animals in a single assay. The output behavior is either scored as an endpoint assay (often reported as a chemotaxis index) or tracked and assessed while the behavior is ongoing, thereby allowing a description of how an animal alters its locomotor behavioral strategies to respond to a stimulus over time (Brown *et al.* 2013; Husson *et al.* 2013; Tanimoto *et al.* 2017). The responses of single animals can also be assessed and have been used to quantitate avoidance behaviors. These assays typically measure the time for an individual animal to reverse from the aversive stimulus or report the percentage of animals that respond by reversing within a given timeframe (Troemel *et al.* 1995; Hart *et al.* 1999; Hilliard *et al.* 2002).

Changes in intracellular calcium levels are generally accepted as a useful readout for sensory neuron activity and are the most accessible surrogate for electrophysiological experiments in *C. elegans*. However, when interpreting calcium imaging data, as described below, it is important to note that there may be scenarios in which calcium signaling does not directly correlate with neuronal depolarization (Zahratka *et al.* 2015). To report changes in calcium, calmodulin-based fluorescent proteins have been used, including FRET-based “cameleon” (Miyawaki *et al.* 1997; Kerr *et al.* 2000; Suzuki *et al.* 2003; Fukuto *et al.* 2004; Hilliard *et al.* 2005) and single emission circularly permutated GFP proteins (GCaMP and its variants) (Romoser *et al.* 1997; Dana *et al.* 2019). An inverse-type reporter was also recently developed to more reliably quantify a drop in calcium following stimulation (Hara-Kuge *et al.* 2018). Importantly, one needs to be aware that if the reporter sequesters calcium, neurotransmission can be disrupted (Ferkey *et al.* 2007). Other readouts for neuronal activity/regulation include cyclic nucleotides, and cGMP levels can also be recorded (Couto *et al.* 2013; Shidara *et al.* 2017; Woldemariam *et al.* 2019). However, there may be subcellular differences in calcium or cyclic nucleotides, including plasma membrane versus the cell body, as well as differences in the cilia, dendrite, cell body, and axon to be considered (S. Woldemariam and N. L'Etoile, unpublished observations) (Shidara *et al.* 2017). Strains that express GCaMP in the nuclei of each neuron have been used to image the entire neural network in real time (Kato *et al.* 2015).

In addition to changes in calcium levels, opening of other nonspecific cation channels may contribute to membrane depolarization, and this needs to be considered. Thus, electrophysiological recordings provide the highest absolute and time-resolved insights into neuronal activity. Although technically difficult, this method has been used to provide high time resolution insights (Goodman *et al.* 1998, 2012) that include the finding that RMD (Mellem *et al.* 2008), AWA (Liu *et al.* 2018a), ASEL (Shindou *et al.* 2019), and other neurons (Faumont *et al.* 2012) fire action potentials and/or exhibit regenerative plateau potentials. To fill the gap between calcium imaging and electrophysiological recordings, genetically encoded voltage sensory hold promise and porting such sensors as the ASAP3 from mice could pave the way (Villette *et al.* 2019).

Microfluidics-based assays have been very useful for simultaneous recording of behavior and neuronal activity in real time (Albrecht and Bargmann 2011; Larsch *et al.* 2013). Briefly, animals are placed into a microfluidic device made of PDMS bonded to a coverslip and shaped into an arena within which the animals’ behavior can be observed. Within the arena, PDMS posts are arranged to provide an artificial “dirt” substrate that the animals can push against as they swim (Lockery *et al.* 2008). Ports flow buffer and stimulus such that they produce a laminar stream, allowing different spatiotemporal stimulus presentations. Using two cameras, one with a low and the other a high magnification objective, both locomotion and neuronal activity (*e.g.*, calcium transients) can be monitored simultaneously (Larsch *et al.* 2013; Levy and Bargmann 2020).

## Neurons and their contributions to chemosensation

**Figure 1:**
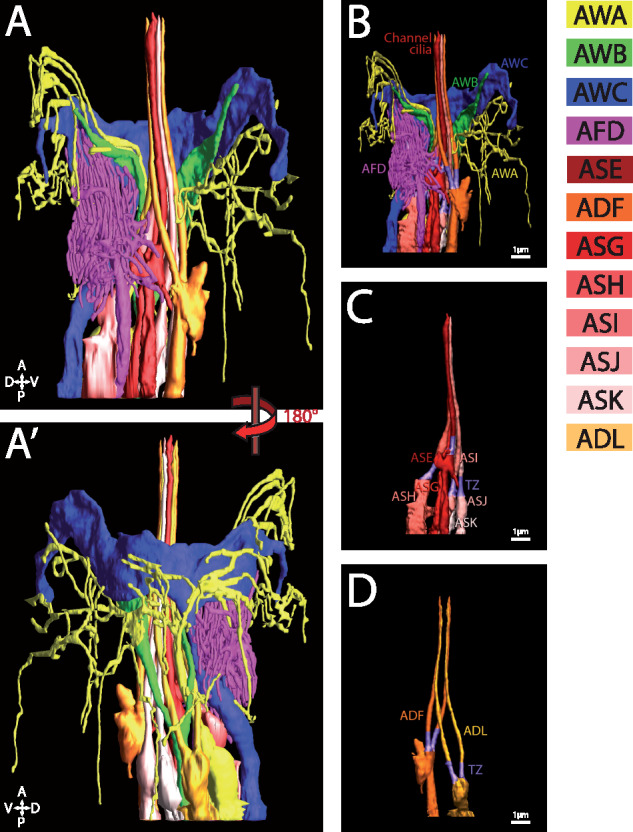
Cilia of amphid sensory neurons. (A and A′) 3 D reconstruction model of the sensory endings of 12 amphid neuronal cilia on the right side. Complex sensory endings of the winged cilia of AWA, AWB, and AWC and microvilli of the AFD neurons are shown in (B). Single (ASH, ASG, ASE, ASI, ASJ, and ASK) and double rod-shaped (ADF and ADL) channel cilia are shown in (C) and (D), respectively. Individual amphid neurons are color coded as indicated. Scale bar: 1 µm. Adapted from Doroquez *et al.* (2014).

There are 32 presumed chemosensory neurons in the hermaphrodite *C. elegans* nervous system. They are housed within the head amphid and inner labial organs, as well as the tail phasmid organs, and are directly or indirectly exposed to the environment (Ward *et al.* 1975; Ware *et al.* 1975; Perkins *et al.* 1986; White *et al.* 1986; Bargmann 2006; Inglis *et al.* 2006). An additional pair of amphid neurons (AFD) is thermosensory (Goodman and Sengupta 2019). Male-specific chemosensory neurons are described elsewhere (Barr *et al.* 2018). The functions of the eleven pairs of amphid and two pairs of phasmid neurons have been extensively characterized in the context of chemosensation, and are the focus here. The ADL, ADF, ASE, ASG, ASH, ASI, ASJ, and ASK neurons have simple, rod-like ciliated sensory endings that terminate within a channel formed by glial cells associated with the amphid sensilla. These neurons primarily detect soluble ligands, although ASH and ADL can also detect volatile ligands (Table 1). The AWA, AWB, and AWC amphid neurons embedded within the sheath glial cells also have ciliated sensory endings that are more complex, and these neurons appear to detect primarily volatile chemicals (Table 1). For a high-resolution ultrastructural analysis of the anterior endings of sensory neurons (and glia) see Doroquez et al. (2014) and Figure 1. The PHA and PHB nociceptive neurons in the phasmid sensilla have ciliated endings that terminate in the animal’s tail.

**Figure 2:**
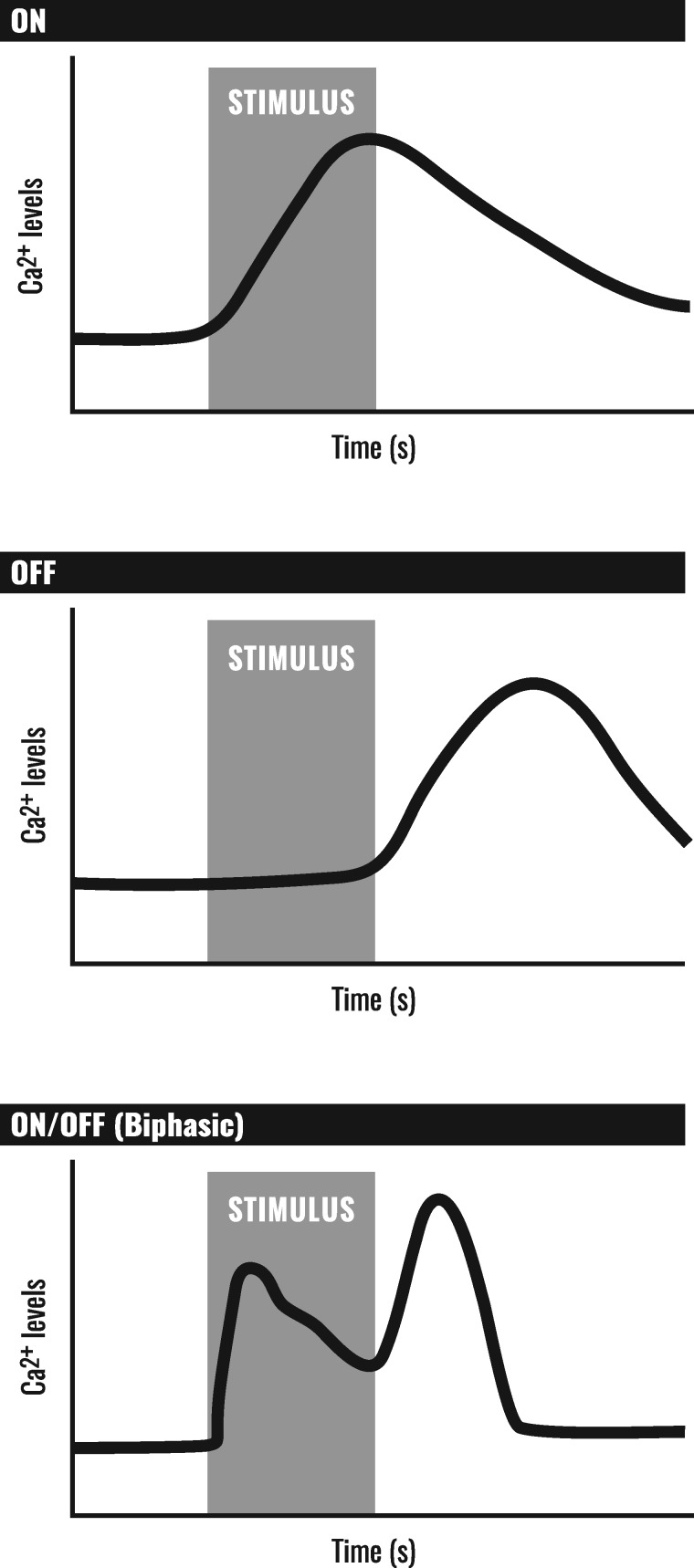
Calcium responses in sensory neurons. Sensory neurons can show a phasic increase in calcium levels upon presentation of stimulus (ON response), an increase in calcium upon removal of stimulus (OFF response), or an increase in calcium upon both the application and again with the subsequent removal of stimulus (ON/OFF or biphasic response).

The majority of examined chemosensory neurons exhibit one of three distinct modes of response to chemical cues: (1) ON responses are *increases* in cytoplasmic calcium presumably due to depolarization that occurs when the concentration of the chemical cue *increases*; (2) OFF responses are *increases* in cytoplasmic calcium that occur when the concentration of the chemical cue *decreases*; (3) ON/OFF (biphasic) responses are *increases* in cytoplasmic calcium that occur in response to both the onset and offset (presentation and removal) of the chemical cue (Figure 2). In this section, we briefly discuss the response physiology, including calcium responses and electrophysiological potentials when known, of the amphid and phasmid chemosensory neurons. A detailed description of signal transduction molecules follows below.

### ASH

The ASH sensory neurons are the main nociceptors in *C. elegans.* These neurons are considered to be “polymodal” because they detect a wide range of aversive stimuli, including both chemical and mechanical cues, similar to nociceptors in systems ranging from other invertebrates such as *Drosophila* (Tracey *et al.* 2003; Zhong *et al.* 2010; Im and Galko 2012; Johnson and Carder 2012) to vertebrates (Besson and Chaouch 1987; Treede 1999; Lee *et al.* 2005). Examples of ASH-detected chemical stimuli are included in (Table 1), and include high concentrations of several odorants that are normally attractive at lower concentrations.

The ASH sensory neurons exhibit a phasic ON response when presented with aversive chemical stimuli; for examples see (Fukuto *et al.* 2004; Hilliard *et al.* 2005; Mills *et al.* 2012; Tanimoto *et al.* 2017; Liu *et al.* 2018b). However, although the ON response appears to be the general rule for ASH, there are also experimental paradigms where an OFF (Thiele *et al.* 2009) or biphasic (ON and OFF) (Chronis *et al.* 2007; Kato *et al.* 2014; Wang *et al.* 2015) response has been observed. Analysis of ASH temporal filter properties suggests that these nociceptors integrate noxious cues over seconds to rapidly reach the activation threshold for avoidance behavior (Kato *et al.* 2014). ASH calcium signaling in response to chemosensory stimuli, and the effects of genetic mutations on it, are discussed extensively in the signal transduction section below. See also (Mirzakhalili *et al.* 2018) for additional computational modeling of ASH signaling.

While neuronal calcium flux is widely considered an indirect measure of neuronal activity, calcium transient amplitudes within the soma may not always be predictive of neuronal depolarization and synaptic signaling. For example, exposure to 1-octanol leads to ASH depolarization (Zahratka *et al.* 2015). But, surprisingly, while the neuromodulator serotonin (5-HT) potentiates ASH depolarization and ASH-mediated avoidance of 1-octanol, it actually decreases 1-octanol-evoked ASH calcium responses (Zahratka *et al.* 2015; Williams *et al.* 2018). These data have been interpreted to indicate that 5-HT enhances ASH excitability by suppressing a calcium-dependent inhibitory feedback loop (Williams *et al.* 2018). Thus, calcium signals and depolarization may not always be directly correlated.

### ADL

In addition to their major role in pheromone detection (*Pheromone*), the ADL neurons play a minor role in chemical avoidance such that their contribution to chemical detection is often revealed only when they are ablated in combination with other sensory neurons. Single and multineuron ablation experiments have revealed a role for ADL in detecting several aversive stimuli (Table 1). In addition, ADL displays an ON response to repellent *P. pacificus* predator cue (Liu *et al.* 2018b). However, although neuronal ablation studies implicate ADL in 1-octanol avoidance (Troemel *et al.* 1995, 1997; Chao *et al.* 2004), ADL does not shows a change in calcium levels following 1-octanol exposure (Mills *et al.* 2012). It is possible that ADL does not respond directly to 1-octanol, or perhaps ablation of ASH causes compensatory changes in ADL and AWB (see below) responsiveness (Mills *et al.* 2012).

### AWB

The AWB neurons detect volatile aversive chemicals. They are the primary mediators of 2-nonanone avoidance (Troemel *et al.* 1997) and play a minor role in the avoidance response to several other odorants (Table 1). Calcium imaging experiments revealed that AWB can respond to distinct stimuli in a variety of ways. For example, while they showed an ON response when presented with 50 mM NaCl (Zaslaver *et al.* 2015), these neurons are activated upon removal of 2-nonanone (OFF response) (Ha *et al.* 2010; Tanimoto *et al.* 2017). Similarly, AWB showed an OFF response upon removal of high-isoamyl alcohol (Yoshida *et al.* 2012) or removal of an *Escherichia coli* supernatant (Zaslaver *et al.* 2015). They also showed an unexpected ON/OFF biphasic response to a low concentration of isoamyl alcohol (10^−4^), which may be related to their possible (very minor) contribution to chemotaxis toward this odorant (Yoshida *et al.* 2012). Similar to ADL (above), 1-octanol exposure/removal did not elicit AWB calcium transients (Mills *et al.* 2012), although ablation studies suggest a minor role for AWB in 1-octanol avoidance (Troemel *et al.* 1997; Chao *et al.* 2004).

### ASK

The ASK neuron pair was first shown to play a minor role in chemotaxis toward the amino acid lysine (Bargmann and Horvitz 1991a). Although it is unusual for a *C. elegans* sensory neuron to detect both attractive and aversive stimuli, ASK also contributes to the avoidance of several soluble stimuli, including SDS (Table 1). Interestingly, ASK showed an OFF response to lysine, but an ON response to SDS (Wakabayashi *et al.* 2009). Because ASK activation promotes reversals (Wakabayashi *et al.* 2004; Gray *et al.* 2005), suppression of calcium signaling by a chemoattractant and activation by a chemorepellent could both contribute to appropriate behavioral responses and locomotion strategies in complex chemosensory environments. For example, calcium imaging revealed that inhibition of ASK by the addition of diacetyl contributes to the disinhibition of the downstream interneuron AIA, allowing AIA to more reliably respond to diacetyl-evoked depolarization of AWA (Dobosiewicz *et al.* 2019). While the application of *E. coli* supernatant decreases ASK calcium levels, an elevation (OFF response) was seen upon its removal (Zaslaver *et al.* 2015). Similarly, an OFF response was also observed with removal of large (but not small) concentrations of suspended bacteria (Calhoun *et al.* 2015). ASK also contributes to pheromone detection (*Pheromone*).

### AWA

The AWA olfactory neuron pair senses bacterially produced volatile cues to direct animals toward potential food sources (Bargmann *et al.* 1993; Larsch *et al.* 2013; Choi *et al.* 2016; Worthy *et al.* 2018a; Dobosiewicz *et al.* 2019; Table 1). However, there are sex differences in attraction to some odorants, including diacetyl (Lee and Portman 2007; White *et al.* 2007; Ryan *et al.* 2014; Barr *et al.* 2018).

AWA is an ON neuron that shows an elevated calcium levels in response to increases in diacetyl, pyrazine, 2-methylpyrazine, 2,4,5-trimethylthiazole and hexyl acetate (Shinkai *et al.* 2011; Larsch *et al.* 2013, 2015; Zaslaver *et al.* 2015; Itskovits *et al.* 2018; Liu *et al.* 2018a; Dobosiewicz *et al.* 2019). This neuron pair also shows an increase in calcium in response to the addition of *E. coli* supernatant, and a decrease in calcium upon its removal (Zaslaver *et al.* 2015). Activated AWA neurons signal to first order interneurons such as AIA that reduce turning probability, thereby elongating runs when an animal heads up the concentration gradient of an attractive chemical (Larsch *et al.* 2015).

As a food sensor, AWA's ability to detect volatiles in gradients that span large concentration ranges is likely to be important for an animal's survival. Indeed, the response properties of AWA enable animals to respond to odorants over a 100,000-fold range of concentrations (*e.g.*, from as low as 11 nM up to 115 mM diacetyl) (Bargmann *et al.* 1993; Larsch *et al.* 2013). Calcium imaging showed that the AWA neurons themselves respond reliably over the same wide span of concentrations (Larsch *et al.* 2013, 2015), with oscillatory responses whose maxima remained constant and did not scale with the concentration of the odor the worm was exposed to (Larsch *et al.* 2015; Itskovits *et al.* 2018). Responses of these neurons to diacetyl sensitize rapidly at high concentrations, thereby allowing the AWA neurons to retain response sensitivity over a wide dynamic range. AWA responses also adapt to the rate of change in concentration rather than to the absolute concentration, which allows the animal to seek out odor concentrations that change most rapidly, thus allowing them to progress along the shortest route to an odor source (Itskovits *et al.* 2018). Interestingly, the oscillations of the left and right AWA neurons were anti-correlated, but between the two they exhibited calcium transients at each upstep of odor (Itskovits *et al.* 2018).

Electrophysiological recordings provided additional insights into how AWA may respond to odors over a broad dynamic range. These studies indicated that AWA fires bursts of 5–20 spikes in about 15% of trials, and these have some of the hallmarks of an action potential (Liu *et al.* 2018a); they are self-limiting, rising sharply then falling to a steady baseline, and they regenerate to recur as a train of spikes (Bean 2007). By imaging GCaMP while injecting current, an algorithm was trained to use the electrophysiological recording to detect spikes within the GCaMP traces. Applying this algorithm to GCaMP traces obtained when the AWA neurons were responding to intermediate concentrations of diacetyl uncovered spiking calcium signals; changes in diacetyl concentration elicited a similar spiking regime as seen with current injections (Liu *et al.* 2018a).

Electrophysiological investigations of AWA also revealed aspects of their responses that indicate how the neurons allow animals to ignore noise, either in the environment or generated by the animal's movement. The time threshold for AWA activation was long, about 300 ms, such that only stimuli that lasted for longer than a third of a second were able to trigger spiking (Liu *et al.* 2018a). This time lag was also sufficient to filter out changes in concentration that would be generated by the typical frequency of head swings generated by self-movement. This ability to filter out noise could be attributed to as yet unidentified potassium channels that increase the resistance of the AWA membrane and keep small fluctuating stimuli from depolarizing the cell (Liu *et al.* 2018a).

The calcium spikes generated by the AWA neurons adapt to the magnitude of the change in odor concentration over time (Liu *et al.* 2018a). Thus, turns should decrease as a function of an increase in odor concentration. However, because AWA activity is discontinuous, rather than directing uninterrupted runs, a decrease in AWA activity is predicted to allow turns to emerge even as an animal climbs a gradient (Itskovits *et al.* 2018). Thus, to model robust climbing of a gradient at higher odor concentrations, the spiking ON neuron pair had to be complemented with OFF neurons that had graded responses (Itskovits *et al.* 2018). The AWC neurons, with their response to intermediate concentrations of diacetyl, may fulfill this role (Dobosiewicz *et al.* 2019).

### AWC

Many attractive odors are sensed by the paired AWC neurons (Table 1), which along with the AWA neurons are the main olfactory neurons in *C. elegans* (Bargmann *et al.* 1993). The two AWC neurons are not symmetric, as they express different G protein-coupled receptors (GPCRs) (Troemel *et al.* 1997; Bauer Huang *et al.* 2007; Vidal *et al.* 2018) and respond to different odorants (Table 1). Odorant bouquets from nutritive bacteria have been found to include known AWC-detected attractive volatiles (Worthy *et al.* 2018a). Some attractive chemicals are also released by nematophagus fungi (Hsueh *et al.* 2017) and pathogenic bacteria (Worthy *et al.* 2018b), which may coopt AWC-mediated attraction to lure *C. elegans* (Zhang *et al.* 2016). These normally attractive odors can become repulsive when worms are sickened or starved in their presence (Tsunozaki *et al.* 2008; Jin *et al.* 2016; Kaletsky *et al.* 2018). The AWC neurons still sense these chemicals under these conditions, but they instead direct repulsion (Tsunozaki *et al.* 2008; Jin *et al.* 2016).

Calcium imaging showed that both AWC neurons are OFF neurons (Chalasani *et al.* 2007, 2010). They are tonically active in buffer, showing low but constant activity that is silenced upon odor addition. Conversely, when odor (or *E. coli* supernatant) is withdrawn, both neurons show a sharp rise in calcium (Chalasani *et al.* 2007, 2010; Kato *et al.* 2014; Calhoun *et al.* 2015; Zaslaver *et al.* 2015; Cho *et al.* 2016; Hsueh *et al.* 2017; Hara-Kuge *et al.* 2018). The AWC neurons induce turns when they are active and forward runs when they are silent (Gray *et al.* 2005; Larsch *et al.* 2013; Gordus *et al.* 2015; Itskovits *et al.* 2018; Dobosiewicz *et al.* 2019), thereby directing runs up an attractive odor gradient.

The AWC calcium response to both odor exposure and removal is rapid (less than a second), robust and reproducible (Kato *et al.* 2014). Modeling showed that the speed of the response is sufficiently rapid, relative to head swings, to allow animals to track an odor gradient using the klinotaxis strategy (Izquierdo and Lockery 2010) (see Appendix), and this was experimentally verified using sensory signal transduction mutants (Kato *et al.* 2014). In addition, the response to a decrease in odor is graded such that it scales with both the amount of odor prior to the decrease and to the change in odor concentration (Cho *et al.* 2016). That is, the odor concentration is integrated over time to set the neuron's response threshold such that odor decreases that fall below the set point (Levy and Bargmann 2020) enable reliable gradient tracking.

The AWC neurons respond to some of the same odors as the AWA neurons, including diacetyl and isoamyl alcohol (Chou *et al.* 2001; Larsch *et al.* 2015; Itskovits *et al.* 2018; Worthy *et al.* 2018a). Interestingly, although AWA shows an oscillatory response to gradients of these odors, AWC responds with graded responses such that the AWC calcium signal is directly proportional to the change in stimulating odor concentration (Cho *et al.* 2016; Itskovits *et al.* 2018; Dobosiewicz *et al.* 2019; Levy and Bargmann 2020). When the responses of AWC and AWA are modeled together, they predict that animals are able to climb less continuous gradients more efficiently (Itskovits *et al.* 2018; Dobosiewicz *et al.* 2019). Furthermore, in contrast to a salt gradient, animals in an odor gradient (isoamyl alcohol) run faster up than down the gradient (Albrecht and Bargmann 2011). This also biases their movement toward the peak of the odor stimulus.

**Figure 3:**
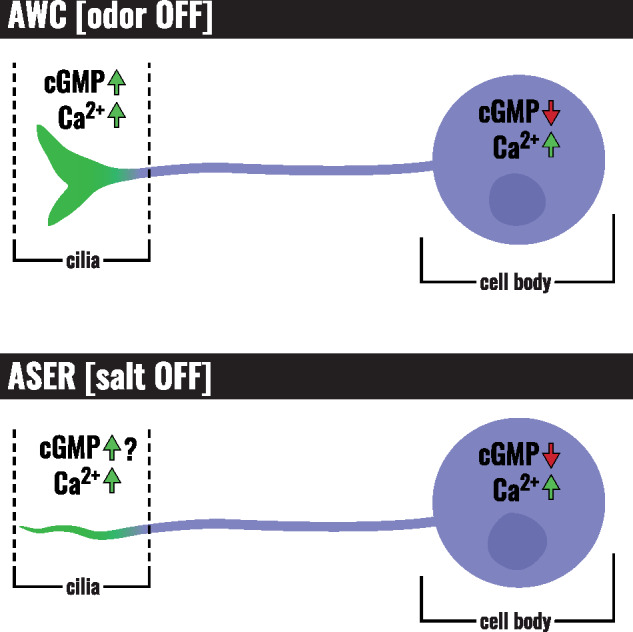
Second messenger levels in cilia versus soma. When odorant is removed from AWC, calcium levels increase in the cilia and the cell body, while cGMP levels increase slightly in the cilia but fall in the cell body. Likewise, when salt is removed from ASER, calcium levels increase in the cilia and cell body, and cGMP levels fall in the cell body. Preliminary data indicate that cGMP levels rise in the ASER cilia when salt is removed (S. Woldemariam and N. L’Etoile, personal communication).

Levels of calcium and cGMP, the primary second messenger in AWC sensory signaling (see below), both initially decrease in the cilia and dendrites in response to onset of odor presentation. But, in the cell bodies, although calcium decreases, cGMP increases with odor onset (Shidara *et al.* 2017; Figure 3). How the cGMP sign is inverted between the cilia and the cell body is unclear, as is the physiological purpose of this inversion.

### ASI

The ASI sensory neurons play an important role in inhibiting entry into the alternative stress-resistant dauer stage under nondauer-inducing conditions (Bargmann and Horvitz 1991b; Schackwitz *et al.* 1996), and are the only source of DAF-7/TGF-β in *C. elegans* grown under standard conditions (Ren *et al.* 1996) (*Pheromone*). The ASIs also play a minor role in chemotaxis to water-soluble stimuli (Table 1), but their contribution is only revealed when ASE (major) and other sensory neurons (minor) are ablated (Bargmann and Horvitz 1991a; Kaufman *et al.* 2005). Ablation studies also showed a role for the ASI neurons in avoidance of worm extract (Zhou *et al.* 2017), SDS and *P. pacificus* predator cue (Liu *et al.* 2018b). They also promote *P. aeruginosa* avoidance, although it is not clear whether this is via direct detection of pathogen-released chemical cues (Cao *et al.* 2017). Calcium imaging experiments revealed that the ASI displays an ON response to HB101 *E. coli* bacteria (Gallagher *et al.* 2013), OP50 *E. coli* bacteria (Calhoun *et al.* 2015) and supernatant (Zaslaver *et al.* 2015), and Luria Broth (LB) (Gallagher *et al.* 2013; Davis et al. 2018), suggesting a role in food sensation. The activation of ASI by external nutrients promotes satiety quiescence (You *et al.* 2008; Gallagher *et al.* 2013). In addition, the aversive stimulus CuSO_4_ elicits an OFF response in ASI that allows them to modulate copper nociception in a reciprocal inhibition circuit with the primary copper detectors, the ASH neurons (Guo *et al.* 2015). *P. pacificus* predator cue also elicits an OFF calcium response in ASI (Liu *et al.* 2018b).

### ADF

The ADF neurons are the only serotonergic sensory neurons in the hermaphrodite (Sze *et al.* 2000) and appear to be tonically active (Thiele *et al.* 2009). Thus, they are uniquely positioned to respond to environmental cues and modulate chemosensory behavioral responses. In the larva, they inhibit entry into the dauer stage under nondauer-inducing conditions (Bargmann and Horvitz 1991b; Schackwitz *et al.* 1996) (*Pheromone*). In adults, under “normoxic” conditions the ADF neurons (along with ASG and ASI) also play a minor role in chemotaxis to water-soluble stimuli (Table 1), but their contribution is only revealed when ASE (major) and other sensory neurons (minor) are ablated (Bargmann and Horvitz 1991a). However, under hypoxic conditions (*e.g.*, those created by high bacterial metabolism in enclosed spaces) the role of ADF (and ASG) in salt chemotaxis may be enhanced due to the upregulation of 5-HT in these neurons (Pocock and Hobert 2010). Calcium imaging experiments have revealed that the ADF neurons show an ON response to *E. coli* supernatant (Zaslaver *et al.* 2015), and they respond directly to repellent levels (1/100) of isoamyl alcohol and indirectly to copper (Shao *et al.* 2019). ADF activation by these stimuli in turn inhibits the ASH nociceptors to modulate aversive chemosensory responses (Shao *et al.* 2019). The ADF neurons also show a calcium ON response to NaCl upsteps, although their activation may not be the result of direct stimulation in this context; ADF may be postsynaptic to a salt-sensitive neuron(s) (Thiele *et al.* 2009).

### ASG

The ASGs play a minor role in inhibiting entry into the dauer stage under nondauer-inducing conditions (Bargmann and Horvitz 1991b; Schackwitz *et al.* 1996). In addition, under ambient (“normoxic”) oxygen conditions, the ASG neurons (along with ADF and ASI) play a minor role in chemotaxis to water-soluble stimuli (Table 1), but their contribution is only revealed when ASE (major) and other sensory neurons (minor) are ablated (Bargmann and Horvitz 1991a). However, under hypoxic conditions the role of ASG (and ADF) in salt chemotaxis may be enhanced due to the upregulation of 5-HT biosynthesis in these neurons (Pocock and Hobert 2010). Surprisingly, in contrast to the cell ablation results, calcium imaging (under normoxic conditions) did not reveal ASG calcium transients in response to either NaCl upsteps or downsteps (Thiele *et al.* 2009; Jang *et al.* 2019). However, the ASG neurons do show spontaneous calcium fluxes independent of salt stimulation, and both the frequency and average size of the activity peaks were higher after salt conditioning under starvation conditions (Jang *et al.* 2019). Thus, via their contribution to switching an animal’s navigation direction relative to a salt gradient, ASG activity may help animals to avoid salt concentrations associated with starvation (Jang *et al.* 2019).

### ASJ

The major role of the ASJ neurons is to regulate dauer entry and exit (*Pheromone*). ASJ promotes dauer formation, such that killing these neurons significantly impaired the ability of wild-type animals to form dauers when exposed to dauer pheromone (Schackwitz *et al.* 1996). ASJ also promotes dauer recovery, and when the ASJ neurons are ablated animals permanently arrest in the dauer stage (Bargmann and Horvitz 1991b). In addition to these roles in the regulation of the dauer state, the ASJ neurons mediate avoidance of *P. aeruginosa*, most likely by detecting both secondary metabolites (Meisel *et al.* 2014) and nitric oxide (Hao *et al.* 2018) produced by these bacteria (Table 1). They also contribute to the avoidance of SDS and *P. pacificus* predator cue (Liu *et al.* 2018b). Calcium imaging experiments revealed that the application of the *P. aeruginosa* secondary metabolite PCN led to an increase in ASJ calcium levels (Meisel *et al.* 2014), as did presentation of 50 mM NaCl, pH 5 or *E. coli* supernatant (Zaslaver *et al.* 2015). Alternatively, an OFF response was seen upon removal of *P. pacificus* predator cue (Liu *et al.* 2018b). ASJ may also play a very minor role in chemotaxis to some water-soluble stimuli (Bargmann and Horvitz 1991a; Kaufman *et al.* 2005).

### ASE

The left and right ASE neurons signal to both shared and distinct interneurons (Cook *et al.* 2019) (see also http://wormwiring.org) and they respond to different chemicals (Table 1). The left and right ASE neurons also express different genes, including receptor guanylyl cyclases (rGCs) that may be tuned to detect these distinct stimuli (Chang *et al.* 2003; Ortiz *et al.* 2009; Smith *et al.* 2013). In addition to this profound difference in sensory function, the two neurons differ in size (subtly) and electrophysiological properties (Pierce-Shimomura *et al.* 2001; Goldsmith *et al.* 2010).

**Figure A1:**
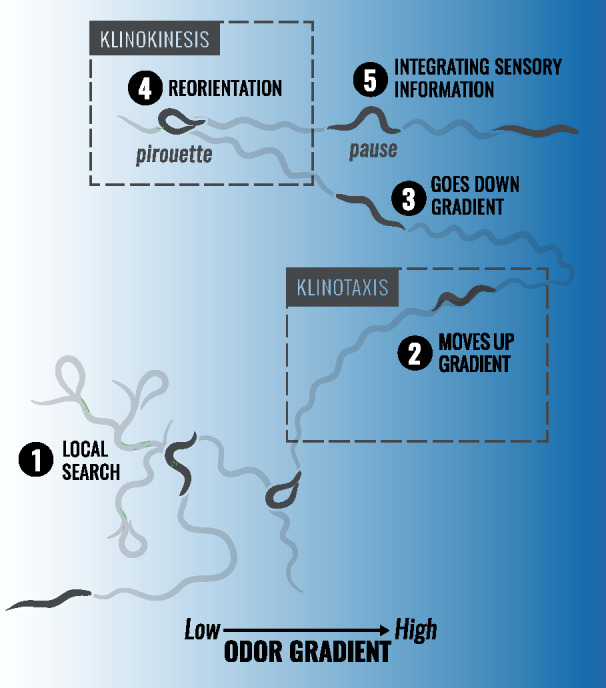
*C. elegans* movement in a gradient. When initially removed from food, the animal executes a search for food in its local vicinity by making short forward movements, backing and making omega turns (1). If the animal senses an attractive chemical gradient (blue) it can run up the concentration gradient and slightly curve into the higher concentration in a movement pattern called klinotaxis (2). If it veers from the gradient and travels to lower concentrations (3), it can reorient by executing a series of reversals and omega turns (termed a pirouette) from which the head is more likely to be reoriented such that it is directed up the gradient (4). Klinokinesis is also known as a biased random walk in which animals run up a gradient, and if they leave the gradient, reorient with turns. This is the major strategy used to travel up the gradient. Animals have been shown to pause (5), which may allow for sensory integration before resuming the search for the source of the attractive chemical cue.

The left and right ASE neurons also differ in their contribution to the locomotor strategies utilized during salt chemotaxis. ASEL responds to an increase in cations and its activity correlates with runs up the gradient, while ASER responds to decreases in anions by initiating pirouettes and decreasing run length (Figure A1). Calcium imaging studies (Pierce-Shimomura *et al.* 2001; Suzuki *et al.* 2008; Kunitomo *et al.* 2013; Luo *et al.* 2014; Wang *et al.* 2017; Lim *et al.* 2018; Shindou *et al.* 2019) and electrophysiology (Shindou *et al.* 2019) corroborate the finding that ASEL is an ON cell that depolarizes and increases intracellular calcium in response to increases in salt concentration (upsteps), while ASER is an OFF cell that depolarizes and increases intracellular calcium with decreases in salt concentration (downsteps). ASEL and ASER respond to changes in salt with a transient influx of calcium that marks the onset of the change (salt up or down, respectively) (Suzuki *et al.* 2008; Oda *et al.* 2011; Luo *et al.* 2014; Lim *et al.* 2018; Shindou *et al.* 2019). This combination of ON and OFF sensory cells underlies the ability of animals to reliably track a smooth gradient, composed of dissolved ion pairs, to its source (Pierce-Shimomura *et al.* 1999, 2001; Suzuki *et al.* 2008; Iino and Yoshida 2009; Izquierdo *et al.* 2015).

Electrical responses to current injection reveal that ASEL and ASER signal in a nonlinear regenerative manner (Goodman *et al.* 1998; Shindou *et al.* 2019) generating plateau potentials (Lockery and Goodman 2009). Responses to current injection depend on extracellular sodium and calcium in concert, but are robust to removal of either alone (Shindou *et al.* 2019). This observation suggests that voltage- and/or calcium-dependent channels underpin nonlinear regenerative signaling. Salt upsteps also evoke plateau potentials in ASEL and the probability of triggering this response is proportional to the change in salt concentration (Shindou *et al.* 2019), providing a mechanism by which ASEL detects and signals the changes in external salt concentration that drive chemotaxis. Additional channels are likely to act in concert with the voltage-gated calcium channel (VGCC) EGL-19 to allow triggering of neurotransmission.

Within ASEL, the salt upstep signal is seen as an influx of calcium in sensory cilium, dendrites, soma and axons (Lim *et al. et al.* 2018; Shindou *et al.* 2019). As described further below, cGMP is the primary second messenger in salt sensory transduction. The cGMP signal at the sensory cilia is translated into changes in intracellular calcium dynamics and further amplified via VGCCs (Shindou *et al.* 2019). Interestingly, although calcium levels increase in the ASEL soma as a result of a salt upstep, cGMP levels decrease (Woldemariam *et al.* 2019). Similarly, ASER somal calcium rises and cGMP falls in response to a salt downstep (Woldemariam *et al.* 2019; Figure 3). However, the mechanism underlying the opposite calcium and cGMP changes in the soma of these neurons is currently unclear.

The ASE neurons also allow an animal to tune its response to salt such that it will become attracted to the salt concentration associated with food experience (Kunitomo *et al.* 2013; Luo *et al.* 2014). Imaging ASEL and ASER calcium levels as the animal is exposed to abrupt downsteps (Kunitomo *et al.* 2013) or is traversing a more natural gradient (Luo *et al.* 2014) revealed that ASER changes the dynamics of its responses to decreases and increases in salt as a function of the salt concentration at cultivation. ASER is most active in response to decreases in salt when the animal is below this set point, driving the animal to higher salt by increasing turning (Kunitomo *et al.* 2013). But, when the animal is at or above the set point and tracks to a lower salt concentration, similar downsteps in salt evoke smaller (Kunitomo *et al.* 2013) and more complex (Luo *et al.* 2014) calcium transients.

### PHA/PHB

The PHA and PHB neurons are located in the phasmid sensory organs of the tail of *C. elegans*, and their role in chemosensation was first shown in 2002 (Hilliard *et al.* 2002). Although ablation of PHA and PHB did not affect SDS avoidance, their ablation in combination with ASH (or ASH and ASK) leads to a stronger avoidance response than ablation of ASH alone (or ASH and ASK) (Hilliard *et al.* 2002). This suggested that PHA/PHB antagonize SDS avoidance that is mediated by the amphid neurons (Table 1), and that the decision to initiate backward locomotion (reversal) is based on the integration of sensory information from the head and the tail (Hilliard *et al.* 2002; Oren-Suissa *et al.* 2016). Shared connections with command interneurons in hermaphrodites further support this model (White *et al.* 1986) (and wormwiring.org). In addition, PHA and PHB also mediate avoidance of dodecanoic acid presented to the tail (Tran *et al.* 2017).

Calcium imaging experiments have shown that PHA and PHB act as polymodal nociceptors, with an ON response to SDS, aversive odors (1-octanol), high isoamyl alcohol, alkaline pH (12), high osmolarity and harsh touch (Zou *et al.* 2017). For each of these stimuli, the responses of PHA and PHB were similar (Zou *et al.* 2017). cGMP imaging of PHB also indicated that SDS triggers an increase in cGMP (Woldemariam *et al.* 2019), which could drive the opening of cyclic nucleotide-gated (CNG) channels that function in the phasmids (Hilliard *et al.* 2002). In contrast, the application of copper decreased calcium levels in PHA/PHB, while copper removal led to an increase in calcium levels (OFF response) (Zou *et al.* 2017). However, while the decrease in calcium signaling appears to be cell autonomous, the OFF response was abolished in *unc-31* mutant animals lacking neuropeptidergic signaling, suggesting that PHA/PHB may be, in part, postsynaptically activated by copper removal via neuropeptides (Zou *et al.* 2017). No calcium transients were observed in response to quinine or acidic pH (Zou *et al.* 2017).

## Chemosensory signal transduction molecules

**Figure 4:**
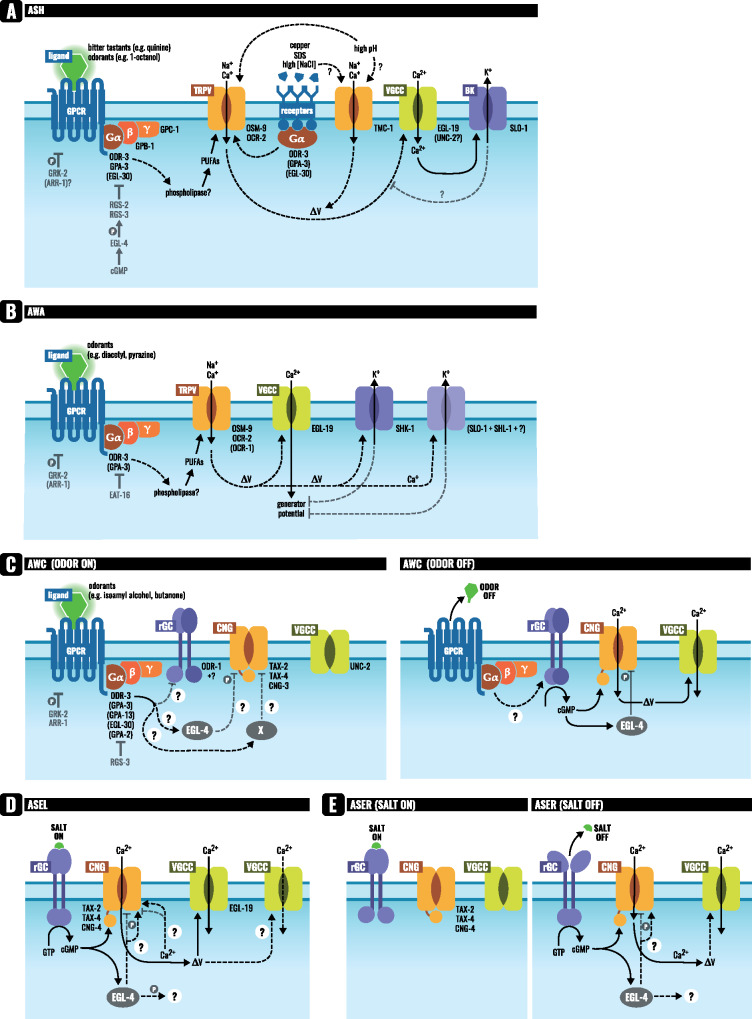
Signal transduction pathways in the ASH, AWA, AWC, and ASE sensory neurons. Simplified models of the potential signal transduction pathways for these representative neurons are shown. See text within the Signal Transduction section for additional details. (A) ASH: Odorant or tastant binding to a GPCR initiates G protein-coupled signaling that likely leads to the generation of PUFAs that activate TRPV channels. Stimuli may also activate other classes of receptors or channels directly. The resulting membrane depolarization activates voltage-gated calcium channels (VGCCs). In a regulatory feedback loop, ASH excitability may be dampened by a calcium-activated potassium channel. Signaling can also be downregulated at the level of GPCRs (via phosphorylation by GRK-2) or at the level of G proteins (by RGS proteins). (B) AWA: AWA signaling is initiated by odorant binding to a GPCR that initiates G protein-coupled signaling that likely leads to the generation of PUFAs that activate TRPV channels. The resulting membrane depolarization can trigger an all or none feed-forward action potential that is generated by opening of the VGCC EGL-19. The amplified voltage change opens voltage-gated potassium channels that subsequently dampen signaling. Signaling is also downregulated by GRK-2 and arrestin, and by an RGS protein. (C) AWC: In the presence of odorant, AWC is silenced. Odorant binding to a GPCR might activate a Gα that inhibits cGMP formation by guanylyl cyclases. The CNG channels may also be inhibited by EGL-4 and possibly by an unidentified protein “X.” Once odor is removed, opening of the CNG channels leads to membrane depolarization that activates VGCCs. Negative regulation of the AWC response occurs via GRK-2 and arrestin, and by an RGS protein. The cGMP-dependent protein kinase EGL-4 likely phosphorylates CNG channels during the adaptation response. (D) ASEL: Signaling is initiated when salt binds to the extracellular domain of the rGC and the intracellular cyclase domains dimerize to cyclize GTP into cGMP. The cGMP produced binds to and opens CNG channels. Membrane depolarization activates VGCCs. EGL-4 is required for calcium signals in response to salt, but its targets (besides TAX-2), and role are unknown. (E) ASER: Salt binding to the extracellular domain of the rGC inhibits cyclase activity and signaling is silenced. Signaling is initiated when salt is removed and the rGC cyclase domains dimerize to cyclize GTP into cGMP, which opens the CNG channel. Membrane depolarization activates a VGCC. Via an unknown mechanism, EGL-4 is required for the calcium flux in ASER.

Below we describe current knowledge about the signaling molecules that transduce chemosensory information within the sensory neurons. We also refer the reader to Hobert (2013) for a broader description of the gene families that function in the *C. elegans* nervous system. While many gene families with neuronal functions appear to be expanded in *C. elegans*, a notable exception is the absence of voltage-gated sodium channels (Bargmann 1998; Hobert 2013). See Figure 4 for a summary of the signal transduction pathways that function specifically within the ASH, AWA, AWC, and ASE neurons.

### G protein-coupled receptors (GPCRs)

The first expression analysis of putative *C. elegans* chemosensory GPCRs was undertaken over 20 years ago (Troemel *et al.* 1995). This foundational study, utilizing the partial genome sequence available, initially identified 41 potential *C. elegans* chemoreceptor genes that fell into six families (*sra*, *srb*, *srg*, *srd*, *sre*, and *sro*) based on sequence similarity with one another (Troemel *et al.* 1995). As completion of the full-genome sequence, a total of approximately 1,300 genes and 400 pseudogenes have been identified, and they are now classified into 19 families (15 of these comprise three major superfamilies: *sra*, *str*, *srg*) (Robertson and Thomas 2006; Thomas and Robertson 2008). Chemosensory GPCR genes are now known to be the largest gene family in *C. elegans*, comprising ∼8.5% of all its genes (Thomas and Robertson 2008). We refer the reader to the primary literature for a more thorough analysis of these gene families and their evolution (Troemel *et al.* 1995; Robertson 1998, 2000, 2001; Chen *et al.* 2005; Thomas *et al.* 2005; Thomas and Robertson 2008; Nagarathnam *et al.* 2012; Krishnan *et al.* 2014).

GFP-based expression analysis of a subset of the first identified putative receptor genes revealed that many were expressed in only a small subset of chemosensory neurons (Troemel *et al.* 1995). In addition, this work established that a single type of chemosensory neuron can express multiple chemoreceptor genes (Troemel *et al.* 1995). This observation has been corroborated multiple times, through studies of individual receptors and sensory neurons, and more recently by a large-scale study that examined the expression pattern of 244 rhodopsin-like (class A) *C. elegans* chemoreceptors (Vidal *et al.* 2018). A small number of *C. elegans* chemosensory GPCRs show left/right asymmetric gene expression, but this asymmetry has so far only been observed for the AWC sensory neuron pair (Troemel *et al.* 1999; Bauer Huang *et al.* 2007; Vidal *et al.* 2018). Consistent with the original findings (Troemel *et al.* 1995), some of the putative chemoreceptors were also found to be expressed in interneurons and motor neurons, and sometimes even in nonneuronal cells (Vidal *et al.* 2018). Thus, it is possible that some receptors may sense internal cues in addition to environmental stimuli. Complementing GFP-based studies with single cell transcriptional profiling (Hammarlund *et al.* 2018) should provide additional insights into the receptor code of individual cells.

In 1996, as the result of behavioral screens for *C. elegans* mutants with specific olfactory defects (odorant-response mutants), ODR-10 became the first odorant receptor in any organism to be paired with its chemical ligand, diacetyl (Sengupta *et al.* 1996). Consistent with a role in detecting environmental stimuli, ODR-10 is localized to the AWA sensory cilia (Sengupta *et al.* 1996), and ODR-10 expression conferred diacetyl responsiveness to other nondiacetyl-sensing neurons and to human HEK293 cells in culture (Zhang *et al.* 1997). Over the years, many groups have attempted to pair additional putative *C. elegans* chemoreceptors with their relevant ligands. However, these efforts have yielded only limited success. This may be due to redundancy among the chemoreceptor genes that sense a particular stimulus, or could suggest that GPCR heteromers are the primary receptors for most chemical stimuli sensed by *C. elegans*. The large size of the *C. elegans* chemoreceptor gene family also makes large-scale candidate gene approaches to de-orphanizing receptors challenging. To date, only six *C. elegans* (nonpheromone) chemosensory receptors have been paired with a chemical ligand (Table 2). Some GPCRs have also been characterized to be pheromone receptors, and these are described separately below (*Pheromone*).

**Table 2:** GPCR and odorant pairings

GPCR	Chemical Ligand	Neurons functioning in	Behavior	Reference(s)
**ODR-10**	Diacetyl (low)	AWA	Attraction	(Sengupta *et al.* 1996) (Zhang *et al.* 1997)
**STR-2**	2-Heptanone	AWC^ON^	Attraction	(Zhang *et al.* 2016)
**DCAR-1**	Dihydrocaffeic acid Benzaldehyde ? (undiluted)	ASH	Avoidance	(Aoki *et al.* 2011)
**SRI-14**	Diacetyl (high)	ASH	Avoidance	(Taniguchi *et al.* 2014)
**SRB-6**	Dodecanoic acid Decanoic acid?	ASH, ADL, ADF (head)^a^ PHA, PHB (tail)	Avoidance	(Tran *et al.* 2017)
**STR-217**	DEET	ADL	“Confusant”	(Dennis *et al.* 2018)

The limited number of *C. elegans* chemosensory GPCRs that have been paired with odorant ligands are shown.

a SRB-6 rescued anterior response when expressed in these three head neurons, but promoters with more restrictive expression patterns were not used (Tran *et al.* 2017).

### G proteins

Heterotrimeric G proteins (comprised of Gα, Gβ, and Gγ subunits) transduce the signals from the transmembrane chemosensory GPCRs to different pathways in different sensory neurons [*e.g.*, see CNG and TRP channels, below]. Briefly, in the classical G protein pathway, when ligand binds to a GPCR a conformational change in the receptor allows it to act as a guanine nucleotide exchange factor (GEF) to facilitate the exchange of GDP for GTP on Gα. Gα-GTP and Gβγ can then activate distinct effectors within the cell (McCudden *et al.* 2005; Weis and Kobilka 2018). The *C. elegans* genome encodes 21 Gα, two Gβ and two Gγ subunits. The complete family of *C. elegans* G proteins, and their roles in diverse processes, have been reviewed previously (Bastiani and Mendel 2006). Here, we focus specifically on the role of G proteins in chemosensory signaling, excluding pheromone responses.

### Gα subunits

*C. elegans* has one clear ortholog of each Gα subunit family: GSA-1 (G_s_), GOA-1 (G_i/o_), EGL-30 (G_q_), and GPA-12 (G_12_) (Lochrie *et al.* 1991; Brundage *et al.* 1996; Park *et al.* 1997; Jansen *et al.* 1999). The remaining 17 *C. elegans* Gα subunits (ODR-3, GPA-1 to GPA-11, and GPA-13 to GPA-17) are somewhat more similar to the G_i/o_ family, but are sufficiently divergent that they are usually referred to as nematode-specific (Roayaie *et al.* 1998; Jansen *et al.* 1999; Jovelin *et al.* 2003; O'Halloran *et al.* 2006). Consistent with a role in sensory signaling, 14 of these (ODR-3, GPA-1, GPA-2, GPA-3, GPA-4, GPA-5, GPA-6, GPA-8, GPA-9, GPA-10, GPA-11, GPA-13, GPA-14, and GPA-15) are expressed in subsets of chemosensory neurons, with individual neurons expressing multiple members of this family (Zwaal *et al.* 1997; Roayaie *et al.* 1998; Jansen *et al.* 1999; Lans *et al.* 2004). Antibody staining revealed that while some Gα subunits (ODR-3 and GPA-13) localize primarily to the sensory cilium of the neurons in which they are expressed, others (GPA-2, GPA-3, and GPA-5) localize to cilia, cell bodies and axons (Roayaie *et al.* 1998; Lans *et al.* 2004). Interestingly, GPA-6 was not found in sensory cilia, but instead was seen in cell bodies and axons (Lans *et al.* 2004). Thus, while some Gαs may be dedicated to transducing signals from chemosensory GPCRs that detect environmental stimuli, others may also interact with GPCRs that respond to internal signals (*e.g.*, neurotransmitters or neuropeptides).

Consistent with localization of ODR-3 in the cilia of the AWA, AWB, AWC, ASH, and ADF head sensory neurons, *odr-3* mutant animals are highly defective for response to most AWA, AWC, and ASH-detected stimuli (Bargmann *et al.* 1993; Roayaie *et al.* 1998; Yoshida *et al.* 2012), and partly defective for response to 2-nonanone (AWB) and quinine (ASH) (Troemel *et al.* 1997; Hilliard *et al.* 2004). The overall relative severity of the *odr-3* mutants suggests that ODR-3 is the primary stimulatory Gα protein that acts downstream of chemosensory receptors in multiple sensory neurons. However, somewhat surprisingly, ODR-3 may also play an inhibitory role in AWB, affecting the time-differential property for sensory input (Tanimoto *et al.* 2017).

ODR-3 also transmits sensory information to influence the behavioral strategies (see Appendix) used during odor tracking. Contributing to their defect in isoamyl alcohol chemotaxis, *odr-3* mutant animals were shown to be defective in klinotaxis throughout a 60-minutes chemotaxis assay using 10^−2^ isoamyl alcohol (Yoshida *et al.* 2012). A defect in klinokinesis (turning) was not observed until after 30 minutes at this concentration, suggesting that other Gα proteins might contribute to proper klinokinesis during the early time period (Yoshida *et al.* 2012). Although both wild-type and *odr-3* animals suppress turning when moving toward isoamyl alcohol and increase turning when moving away from the odor (klinokinesis), *odr-3* mutants curve in the wrong direction when moving away from the odor source (Kato *et al.* 2014). This may be due to altered “active sensing” during forward locomotion (Kato *et al.* 2014). When animals are in a spatial gradient, head swings should result in an oscillation in the odor concentration at the tip of the animal’s nose that guides steering as part of the klinotaxis strategy. However, dynamic analysis of AWC signaling in response to pulses of isoamyl alcohol showed that, in addition to being diminished, the calcium fluxes lag behind odor presentation in *odr-3* mutants (Kato *et al.* 2014). This suggests that ODR-3 normally accelerates the AWC response to short pulses of stimulus, thereby allowing these neurons to actively sense changes in the odor gradient as the animal swings its head (Kato *et al.* 2014).

Because *odr-3* mutant animals do retain at least a residual behavioral response to most stimuli tested, it suggests a role for additional Gα proteins in chemosensory signaling (Bargmann *et al.* 1993; Troemel *et al.* 1997; Roayaie *et al.* 1998; Jansen *et al.* 1999; Hilliard *et al.* 2004; Yoshida *et al.* 2012). Indeed, although individual mutation of most other Gα-encoding genes leads to only subtle effects on chemosensation, double and multi-mutant analyses have revealed both stimulatory and inhibitory Gα signaling roles (Jansen *et al.* 1999; Hilliard *et al.* 2004; Lans *et al.* 2004). For example, while ODR-3 plays a major role in AWA-mediated chemotaxis, GPA-3 also contributes, and GPA-5 plays an inhibitory role (Jansen *et al.* 1999; Lans *et al.* 2004). In the AWC neurons, ODR-3 again acts as the major transducer of chemosensory signals, along with more minor contributions from GPA-3 and GPA-13, while GPA-2 is inhibitory (Lans *et al.* 2004). However, GPA-2 may also contribute to butanone detection (Roayaie *et al.* 1998). The AWC neurons may also use GPA-3 along with EGL-30 to transduce the 2-heptanone signal from the STR-2 receptor (Zhang *et al.* 2016). In response to the ASH and ASK (minor) -detected stimulus quinine, GPA-3 plays a major role and ODR-3 also contributes (Hilliard *et al.* 2004). However, *gpa-3; odr-3* double mutants are completely defective in quinine response. Interestingly, *egl-30* single mutant animals are also partially defective in response to quinine, suggesting an additional role for G_q_ signaling (Esposito *et al.* 2010).

Although ODR-3 is required for ASH-mediated avoidance of high NaCl (Hukema *et al.* 2006), no sensory Gα has been found to play a role in NaCl chemotaxis (Roayaie *et al.* 1998; Hukema *et al.* 2006). Instead, ODR-3 and GPA-1 contribute to salt gustatory plasticity (Hukema *et al.* 2006).

Consistent with behavioral analyses, calcium imaging experiments showed that ASH calcium transients are significantly decreased in *odr-3* mutants in response to five distinct ASH-detected stimuli: copper, glycerol, SDS, quinine (Hilliard *et al.* 2005), and high-isoamyl alcohol (Yoshida *et al.* 2012). In contrast, loss of *gpa-3* alone only decreased calcium signaling in response to quinine, indicating a more repellent-specific role for GPA-3 (Hilliard *et al.* 2005). However, there is a complete loss of the ASH calcium flux in response to copper, glycerol, SDS and quinine in *odr-3; gpa-3* double mutant animals, indicating that GPA-3 does contribute to the response to these other ASH-detected stimuli as well (Hilliard *et al.* 2005). Similarly, while the AWC^ON^ calcium transients of *odr-3* mutants were comparable to wild-type animals, they were dramatically decreased in *odr-3; gpa-3* double mutants (Yoshida *et al.* 2012). Animals lacking GOA-1 function fail to avoid strongly alkaline pH although the calcium influx in ASH is normal, suggesting that GOA-1 functions downstream of the OSM-9/OCR-2 TRPV channels in this context (Sassa and Maruyama 2013). GPA-11 also plays a modulatory role in ASH, acting downstream of 5-HT signaling (Chao *et al.* 2004).

### Gβγ subunits

The two *C. elegans* Gβ subunits are encoded by *gpb-1* and *gpb-2* (van der Voorn *et al.* 1990; Zwaal *et al.* 1996; Jansen *et al.* 1999). GPB-1 belongs to the Gβ_1–4_ subtype that requires Gγ coupling for function (Smrcka 2008). *gpb-1* is a ubiquitously expressed and essential gene, rendering behavioral analysis of global loss-of-function mutant animals infeasible (Zwaal *et al.* 1996). However, neuronally targeted RNAi experiments revealed a role for GPB-1 in chemosensory signaling (Esposito *et al.* 2007; Yamada *et al.* 2009). ASH-selective knock-down of *gpb-1* leads to defective avoidance responses to quinine and high osmolarity (Esposito *et al.* 2007). In addition, GPB-1 acts with the Gγ subunit GPC-2 to promote AWC-mediated chemotaxis to benzaldehyde (Yamada *et al.* 2009). GPB-2 is most similar to the divergent vertebrate Gβ5 subunit, which can interact with the GGL domain of regulator of G protein signaling (RGS) proteins (Smrcka 2008). GPB-2 contributes to benzaldehyde olfactory adaptation (Matsuki *et al.* 2006; O'Halloran *et al.* 2009), most likely via coupling to the RGS protein EGL-10 instead of GPC-1 (Yamada *et al.* 2009). Targeted cell-specific knockouts of *gpb-2* may aid in further characterization of its role in chemosensory signal transduction.

Animals with a loss-of-function mutation in the Gγ-encoding gene *gpc-1* are defective for adaptation to the water soluble attractants (tastants) NaAc, NaCl, NH_4_Cl (Jansen *et al.* 2002), as well as gustatory plasticity in response to NaCl (Hukema *et al.* 2006, 2008). In the ASH nociceptors, loss of GPC-1 function leads to a partially reduced initial calcium transient in response to quinine, but not copper, glycerol, or SDS (Hilliard *et al.* 2005). However, consistent with the main role of GPC-1 being in adaptation, *gpc-1* loss-of-function animals are also defective in sensory adaptation to all four tested ASH repellent stimuli, as assessed by calcium imaging (Hilliard *et al.* 2005). In addition, the Gβ subunit GPB-1 couples to GPC-1 to promote adaptation to benzaldehyde (Yamada *et al.* 2009). Differences in assay format may explain why the *gpc-1* olfactory adaptation defect was not observed previously (Jansen *et al.* 2002).

### Guanylyl (guanylate) cyclases (GCs)

GCs produce cGMP, the soluble messenger that regulates processes as divergent as foraging (*C. elegans*, *D. melanogaster*), learning and memory (*C. elegans*, *R. norvegicus domesticas*), vasodilation and visual signal transduction (mammals) (Osborne *et al.* 1997; Fujiwara *et al.* 2002; Sharma *et al.* 2016). cGMP gates the opening of CNG channels, activates cGMP-dependent protein kinases and [in most animals besides nematodes (Hobert 2013)] cyclic nucleotide-hyperpolarizing channels, and activates phosphodiesterases that ultimately degrade cGMP. GCs exist in two forms: soluble cyclases that are activated by gaseous stimuli and are not further discussed in this chapter, and the rGCs that have a transmembrane domain and can transduce gas, environmental chemical, peptide and thermal signals (Yu *et al.* 1997; Hallem *et al.* 2011; Maruyama 2017; Goodman and Sengupta 2018). rGCs can act downstream of G protein-coupled receptors via activation by Gαs and/or they can be directly regulated by ligand binding to or detachment from their extracellular domains.

*C. elegans* expresses 27 rGCs (Yu *et al.* 1997; Fitzpatrick *et al.* 2006; Ortiz *et al.* 2006) and all are found in sensory neurons, except GCY-11, which is expressed in pharyngeal muscle. Nearly half (11/27) are expressed in the gustatory ASE neurons (Ortiz *et al.* 2006), while the rest are expressed in other sensory neurons that also express CNG channels: ADL, AWB, AWC, ASG, ASI, ASJ, ASK, AFD, AQR, PQR, URX, PHA, and PHB, as well as a few interneurons and nonneuronal cells. Members of the large *C. elegans* rGC family show great heterogeneity in their extracellular ligand binding domains (Fitzpatrick *et al.* 2006). The expansion of this feature may reflect the evolutionary pressure this organism has experienced to sense and respond to a wide variety of ligands via guanylyl cyclase receptors, and may allow animals to respond to environmental stimuli that do not typically interact with GPCRs. Here, we focus on the role of rGCs in chemosensory signaling and also recommend this review (Maruyama 2017). See the Appendix for discussion of rGC structure and activation mechanisms, including homo- and hetero-dimer formation. Briefly, each rGC is a dimer of two polypeptides, each encoding a half-cyclase domain. Cyclase activity of the dimer requires that the half-cyclase domains come together to form an active enzyme that cyclizes cGMP from GTP. This dimerization can be regulated by ligand binding to the receptor domain, phosphorylation, or regulatory protein binding to the intracellular domains (ICDs) (Sharma *et al.* 2016).

### rGCs and their chemosensory functions

**ODR-1** and **DAF-11** mRNA are co-expressed in AWC, AWB, ASI, ASJ, and ASK (Birnby *et al.* 2000; L'Etoile and Bargmann 2000) [http://www.cengen.org, (Hammarlund *et al.* 2018)]. Although an ODR-1::GFP fusion expressed from a multi-copy transgene was expressed in AWC, AWB, ASI, ASJ and ASK, a CRISPR-edited GFP-tagged ODR-1, is expressed only in AWC and AWB under standard laboratory conditions (B. Zhang, V. Paketci, C. Zuazo, B-T. Juang and N. L'Etoile, unpublished observations).

Both ODR-1 and DAF-11 are required for AWC-mediated chemotaxis to isoamyl alcohol, benzaldehyde and butanone, and for AWB-mediated repulsion from 2-nonanone (Birnby *et al.* 2000; L'Etoile and Bargmann 2000), and thus they were posited to act as heterodimers (Morton 2004; Ortiz *et al.* 2006). Indeed, DAF-11 and ODR-1 are both required downstream of the GPCR LITE-1 to mediate the response to light (Liu *et al.* 2010). However, there are also clues that they could act as homodimers as well as heteromers. For example, ODR-1 is exquisitely localized to the AWC cilia, while DAF-11 is expressed throughout the cell (B. Zhang, V. Paketci C. Zuazo and N. L'Etoile, unpublished observations) (Birnby *et al.* 2000; L'Etoile and Bargmann 2000). Loss of the cGMP-dependent protein kinase EGL-4 suppresses the benzaldehyde chemosensory defects of *daf-11* mutants, but fails to suppress *odr-1* defects (N. L'Etoile and C. Bargmann, unpublished results) (L'Etoile *et al.* 2002). Evidence from cGMP imaging also indicates that the drop in cGMP in AWC when odor is applied requires ODR-1, and only partially depends on DAF-11 (Shidara *et al.* 2017). The reduction in cGMP may be a consequence of negative regulation of ODR-1, perhaps by a phosphorylation of the kinase-like region, binding of a negative regulator to the hinge region, or by inhibition by a Gα protein.

In addition, although DAF-11 is required in ASJ and possibly ASK to block dauer formation (Schackwitz *et al.* 1996), *odr-1* mutants do not show dauer phenotypes (L'Etoile and Bargmann 2000). Furthermore, although DAF-11 and GCY-27 are both required in ASJ for response to nitric oxide, they are unlikely to act as heteromers with each other in this context, as DAF-11 is required for the ON response and GCY-27 for the OFF response (Hao *et al.* 2018).

**GCY-1, GCY-4,** and **GCY-22** act in ASER (Smith *et al.* 2013), the sensory neuron that promotes chemotaxis to the salt concentration last associated with food (Kunitomo *et al.* 2013; Luo *et al.* 2014). The GCY-22 ECD directs Cl^−^, I^−^, Br^−^ and methionine seeking responses when appended to the ICDs of GCY-1 or GCY-4, and co-expressed in ASI in *gcy-22* mutant worms (Smith *et al.* 2013). Surprisingly, imaging experiments in ASER showed that GCY-22 is required for both the calcium increase in response to Cl^-^ removal, and paradoxically, the cGMP decrease in response to Cl^−^ removal (Ortiz *et al.* 2009; Woldemariam *et al.* 2019). Furthermore, the ECD of GCY-1 is required for specific recognition of K^+^ ions, and the ECD of GCY-4 for I^−^ (Smith *et al.* 2013).

**GCY-14** is localized to the ASEL cilia and is required both for chemotaxis to Na^+^ and Li^+^ ions (Ortiz *et al.* 2006) and for the response of ASEL to high pH (acting as a homodimer in this case, as shown by second site suppressor mutagenesis) (Murayama *et al.* 2013). Mis-expression of GCY-14 (in the ASI neurons) was sufficient to confer calcium responses to alkaline pH in *gcy-14* mutants, and a pH-sensitive histidine residue in its ECD was required to signal the increase in extracellular pH (although it was not required for the response of this rGC to Na^+^) (Murayama *et al.* 2013). Thus, GCY-14 is likely directly stimulated by pH upsteps (increases) to produce cGMP, with hydroxyl ions acting as the likely ligand that binds to its ECD, triggering a cascade of changes that result in dimerization and activation of the cyclase. Increased cGMP could then open CNG channels in ASEL to promote runs toward the stimulus.

**GCY-27** is required in ASK, and perhaps ASH, to decrease ASH-mediated aversion of bitter tastants (Krzyzanowski *et al.* 2013). GCY-27 has a very short ECD, so it may only respond to intracellular ligands or, if it transduces extracellular signals, it may act as a heterodimer to do so.

**GCY-12** is required to regulate an animal's body size. It is expressed in ASE and AWC, and its ECD is dispensable for body size regulation (Fujiwara *et al.* 2015). A possible role for this enzyme in chemosensation has yet to be described.

**GCY-28** is expressed in the axons of AWC where it is required for the butanone exposure-induced switch from attraction to repulsion after prolonged starvation. It appears to act in the AWC axons, where it may affect synaptic transmission (Tsunozaki *et al.* 2008).

### Phosphodiesterases (PDEs)

PDEs degrade cGMP and thus are crucial for regulating signaling. In vertebrate photoreceptors, rhodopsin activation by light activates PDEs that degrade cGMP, thereby decreasing the open probability of CNG channels (Fu and Yau 2007). Signaling by some chemical stimuli may similarly require rapid degradation of cGMP. *C. elegans* expresses six PDEs: PDE-1, PDE-2, PDE-3, PDE-4, PDE-5, and PDE-6 (Liu *et al.* 2010). The PDE-4 and PDE-6 proteins are homologous to human PDEs that have specificity for cAMP over cGMP (Liu *et al.* 2010). The remaining PDEs are most similar to those that can cleave both cAMP and cGMP (Omori and Kotera 2007). PDE-1 has a calcium regulatory domain and degrades cGMP in response to calcium increases (Couto *et al.* 2013). Other PDEs, such as PDE-2, are activated by cGMP and are thus capable of providing negative feedback and stabilization of cGMP levels (Couto *et al.* 2013; Rahi *et al.* 2017). Thus far, no *C. elegans* PDE has been shown to play a direct role in regulating chemosensory signaling, although PDE-1, -2, -3, and -5 are involved in adaptation to odor stimuli (O'Halloran *et al.* 2012).

### Cyclic nucleotide-gated (CNG) channels

CNG cation channels, whose open probabilities are increased by the binding of cGMP or cAMP to intracellular cyclic nucleotide binding domains, play key roles as primary sensory channels in phototransduction and olfaction across species (Pifferi *et al.* 2006). They are localized to sensory endings where their opening/closing generates a change in membrane potential following the delivery of a chemosensory stimulus, while other voltage-gated channels that are expressed more widely in the neuron may amplify the signal (Shindou *et al.* 2019). Functional CNG channels are tetramers, composed of one to four A-type (alpha) and a variable number of B-type (beta) subunits arranged around a central pore (Pifferi *et al.* 2006). Subunit types are identified by amino acid residues within their pore domains that determine ion selectivity (Root and MacKinnon 1993; Eismann *et al.* 1994; Seifert *et al.* 1999), as well as the presence (A-type) or absence (B-type) of a leucine zipper in their C-termini (Zhong *et al.* 2002; Shuart *et al.* 2011).

The *C. elegans* A-type (TAX-4) (Komatsu *et al.* 1996, 1999) and B-type (TAX-2) (Coburn and Bargmann 1996; Coburn *et al.* 1998) subunits have close mammalian homologs (L’Etoile 2004; Wojtyniak *et al.* 2013), while the less conserved subunits (CNG-1 and CNG-3: A-types; CNG-2 and CNG-4/CHE-6: B-types) are much more diverged (Cho *et al.* 2004, 2005; L’Etoile 2004; Smith *et al.* 2013; Wojtyniak *et al.* 2013). *In vitro* experiments showed that a channel's affinity for cGMP, as well as how long it stays open once it binds cGMP, depends on which subunits comprise the channel (Komatsu *et al.* 1999; O'Halloran *et al.* 2017). For example, homomeric channels comprised of only TAX-4 (A-type) have a 10-fold higher affinity for cGMP and stay open seven times as long as a channel comprised of both TAX-4 (A-type) and TAX-2 (B-type) subunits (Komatsu *et al.* 1999; O'Halloran *et al.* 2017). Addition of other (diverged) A- or B-type subunits to the channel also changes its biophysical properties and this is important for function (O'Halloran *et al.* 2017). The subunit composition of each channel also dictates which subdomain of the sensory cilia the CNG channel resides within, and the subdomain each channel occupies is specific to each sensory neuron (Wojtyniak *et al.* 2013). Thus, the specific function each sensory neuron serves may require distinct regions of its sensory cilia to respond to cGMP with different dynamics and sensitivity.

Consistent with TAX-2 being a core component of many CNG channels, and its expression pattern (AWC, ASE, ASG, ASI, ASJ, ASK, AWB, AFD, ADE, and BAG), *tax-2* mutant animals are defective for a variety of sensory responses, including chemotaxis toward the AWC-detected odorants isoamyl alcohol and benzaldehyde (Coburn and Bargmann 1996) and in AWB-mediated avoidance (Troemel *et al.* 1997; Yoshida *et al.* 2012). They are also defective in lysine chemotaxis (Coburn and Bargmann 1996). TAX-4 has a similar expression pattern (AWC, ASE, ASG, ASI, ASJ, ASK, AWB, AFD, BAG, and URX), and *tax-4* mutant animals are also defective for chemotaxis toward isoamyl alcohol, benzaldehyde and 2-butanone (also detected by AWC), and partially defective for 2,4,5-trimethylthiazole (detected by AWA and AWC) (Komatsu *et al.* 1996). In addition, TAX-4 contributes to PHA/PHB-mediated avoidance of SDS (Hilliard *et al.* 2002). TAX-4 can also act in a TAX-2 independent manner, as evidenced by the finding that TAX-4, but not TAX-2, is required in ASI and ASJ to respond to sulpholipid cues secreted by the predator *P. pacificus* (Liu *et al.* 2018b).

Both *tax-2* and *tax-4* mutants are defective in ASE-mediated NaCl chemotaxis (Coburn and Bargmann 1996; Komatsu *et al.* 1996), ASEL-mediated chemotaxis toward alkaline pH (Murayama *et al.* 2013), and ammonium sensation (most likely mediated by AWC) (Frøkjær-Jensen *et al.* 2008). They are both defective in preferring the smell of *P. aeruginosa* PA14 over *E. coli* OP50 bacteria (Harris *et al.* 2014), *S. marcescens* avoidance (Pradel *et al.* 2007), *Microbacterium nematophilum* avoidance (Yook and Hodgkin 2007; Anderson and McMullan 2018) and worm extract avoidance (Zhou *et al.* 2017).

Imaging experiments revealed that the TAX-2 and TAX-4 subunits are required for the ASEL calcium flux in response to an NaCl upstep (Suzuki *et al.* 2008) and to a pH upstep (6.8 to 10) (Murayama *et al.* 2013). TAX-2 and TAX-4 are also required for the ASER calcium flux in response to an NaCl downstep (Suzuki *et al.* 2008), and TAX-4 contributes to isoamyl alcohol sensing in PHA/PHB (Zou *et al.* 2017).

CNG channels generate calcium fluxes that can ultimately regulate gene expression. For example, loss of either TAX-2 or TAX-4 perturbs asymmetric expression of STR-2 in AWC (Troemel *et al.* 1999). Both channel subunits are also necessary to transmit signals that induce *daf-7* expression in the ASJ neurons and increase its expression in ASI neurons when animals are cultured on PA14 (Meisel *et al.* 2014). They are required for attraction to 2-heptanone, but their role in this context may be maintenance of STR-2 receptor expression in AWC^ON^ rather than in transducing the olfactory signal (Zhang *et al.* 2016). In addition, both TAX-2 and TAX-4 help to promote and prevent dauer formation, in different contexts, depending on the neuron they are expressed in (*Pheromone*) (Coburn *et al.* 1998). In addition to their similar expression patterns and loss-of-function phenotypes, electrophysiological data also suggest that TAX-2 and TAX-4 can form heteromeric channels (Komatsu *et al.* 1999; O'Halloran *et al.* 2017). However, complex mixtures of homomeric and heteromeric channels are likely to be expressed in sensory neurons (Wojtyniak *et al.* 2013).

CNG-1 is expressed in unidentified head neurons (but including ASI) and PHA/PHB in the tail (Cho *et al.* 2005; Wojtyniak *et al.* 2013). Although *cng-1* mutant animals showed no defect in olfaction (AWC or AWA-mediated) or NaCl chemotaxis (Cho *et al.* 2005), CNG-1 is required for starvation-induced sharpening of the response to odors that are sensed by both AWC^ON^ and AWC^OFF^ (He *et al.* 2016). It may also regulate sensory integration in the AIA interneurons (Shinkai *et al.* 2011).

CNG-2 is expressed in just a subset of the cells that express TAX-2/TAX-4: AWC, ASE, ASG, ASI, ASJ, and ASK (Wojtyniak *et al.* 2013). CNG-2 is required for the PA14 metabolite-induced calcium flux and *daf-7* induction in ASJ (Park *et al.* 2020), but its function in the remaining neurons is not known. However, it may be involved in plasticity induced by cGMP and calcium signaling, as it possesses consensus cGMP-dependent protein kinase (PKG) phosphorylation and calmodulin binding sites.

CNG-3 expression is also restricted to a subset of the cells that express TAX-2/TAX-4: AWC, ASE, ASI, AWB, and AFD (Cho *et al.* 2004; Wojtyniak *et al.* 2013). However, despite being expressed in chemosensory neurons, *cng-3* mutant animals showed no defect in chemotaxis to AWC (or AWA) detected odorants, or NaCl chemotaxis (Cho *et al.* 2004; O'Halloran *et al.* 2017). Instead, it plays a role in short-term (30 minutes), but not long-term (>60 minutes), adaptation of AWC to the attractive odorants benzaldehyde and 2-butanone (O'Halloran *et al.* 2017). Indeed, CNG-3 may be regulated by both cGMP and calcium signaling because it has a consensus PKG site at serine 20 that is required for adaptation to a 30 minutes exposure of AWC-sensed odors (O'Halloran *et al.* 2017) and a putative calmodulin binding site at L551-L565. Biomolecular Fluorescence Complementation (BiFC) assays suggested that CNG-3/TAX-2 and CNG-3/TAX-4 interactions likely occur *in vivo* (O'Halloran *et al.* 2017). In cell culture, the addition of CNG-3 to TAX-2/TAX-4 channels altered their gating kinetics (O'Halloran *et al.* 2017).

CNG-4 expression is very weak, challenging reliable identification of cells beyond AWC and ASE (Smith *et al.* 2013; Wojtyniak *et al.* 2013). Although *cng-4* (also known as *che-6*) mutants show no defects in AWC-mediated olfaction, they are defective in the ASE-mediated response to water soluble attractants (including NaCl, cAMP and biotin) (Bargmann *et al.* 1993; Smith *et al.* 2013).

### Transient receptor potential (TRP) channels

TRP channels are cation channels that are important for responses to many types of external stimuli, including light, sound, touch, temperature, and chemicals (Venkatachalam and Montell 2007; Samanta *et al.* 2018). The *C. elegans* genome encodes 23 TRP channels, including members of each of the seven TRP subfamilies (Goodman and Schwarz 2003; Hobert 2013). For an extensive review of the varied roles of these cation channels in *C. elegans* (see Kahn-Kirby and Bargmann 2006; Bounoutas and Chalfie 2007; Xiao and Xu 2009, 2011; Schafer 2015; Goodman and Sengupta 2019).

To date, only TRPV family members have been shown to play a role directly in chemosensory behavior in *C. elegans*. The TRPV channel OSM-9 was the first TRP channel shown to have a role in invertebrate chemosensation and was the first TRP channel to be functionally characterized in *C. elegans* (Colbert and Bargmann 1995; Colbert *et al.* 1997). Sequence analysis lead to the subsequent identification of four additional *C. elegans* TRPV family members, *ocr-1*, *ocr-2*, *ocr-3*, and *ocr-4* (*osm-9*/capsaicin receptor related) (Tobin *et al.* 2002).

Transcriptional and translational reporters expressed from extrachromosomal arrays indicate that OSM-9 can be expressed in multiple sensory neurons, including the AWA, AWC, ASH, ADL, ASE, ADF, ASG, ASI, ASJ, and ASK head chemosensory neurons, as well as PHA and PHB in the tail (Colbert *et al.* 1997), while the single cell transcriptional profiling dataset [http://www.cengen.org, (Hammarlund *et al.* 2018)] indicates that the mRNA is most highly expressed in AWA, ADF, ADL, ASH, OLQ, PQR, PHA, and PHB. A CRISPR-edited GFP-tagged OSM-9 confirms the single cell sequencing dataset (K. Benedtti, F. Saifuddin, N. L’Etoile, personal communication). While there are a number of neurons that may express *osm-9* (Colbert *et al.* 1997), but not any of the *ocr* genes (including AWC, ASE, ASG, ASI, ASJ, and ASK) (Tobin *et al.* 2002), each *ocr* gene is only expressed in a subset of cells that express OSM-9. This suggests that individual OCR channel subunits can function together with OSM-9 in distinct contexts. In particular, OCR-2 is also expressed in AWA, ASH, ADL, ADF, PHA, and PHB (Tobin *et al.* 2002). In cells in which OSM-9 and OCR-2 are co-expressed, they are localized to the cilia and are mutually dependent on each other for cilia localization (Tobin *et al.* 2002). This, combined with behavioral data (see below), suggests that these two proteins come together to function in a single channel complex to mediate primary signal transduction. An exception to this is that OCR-2 functions in an OSM-9 independent manner in ASH and ADL to generate avoidance of *P. pacificus* predator cue (Liu *et al.* 2018b). OSM-9 is not required for AWC-mediated sensory responses, and instead is required for adaptation to some AWC-sensed stimuli (Colbert and Bargmann 1995). Its exact role in adaptation is still ambiguous, as it may (Colbert *et al.* 1997) or may not [K. Benedtti, F. Saifuddin, N. L’Etoile, personal communication and http://www.cengen.org, (Hammarlund *et al.* 2018)] be expressed in AWC.*osm-9* and *ocr-2* mutant animals are defective in chemotaxis to odorants detected by the AWA olfactory neurons, such as diacetyl and pyrazine (Colbert *et al.* 1997; Tobin *et al.* 2002). In imaging experiments, *osm-9* single and *ocr-1 ocr-2* double mutant animals showed no AWA calcium flux in response to diacetyl (Larsch *et al.* 2015). The calcium response was also reduced 1000-fold in *ocr-2* single mutant animals (Larsch *et al.* 2015). Thus, OSM-9 and OCR-2 are likely to be the sensory transduction channel downstream of chemical stimulation. Experiments in *osm-9* mutant animals suggest that the TRPV channels also contribute to setting the threshold of AWA electrical excitability (Liu *et al.* 2018a).

Animals lacking OSM-9 or OCR-2 function display diminished responses to a broad range of ASH-detected chemical stimuli, including high benzaldehyde, high pH, 1-octanol, 2-octanone, copper, SDS and bitter tastants (including quinine) (Colbert and Bargmann 1997; Tobin *et al.* 2002; Hilliard *et al.* 2004; Ezak *et al.* 2010; Sassa *et al.* 2013; Wang *et al.* 2015, 2016). OSM-9 and OCR-2 may also contribute to ASH-mediated avoidance of high NaCl (Hukema *et al.* 2006). OSM-9 and OCR-2 also appear to contribute to social feeding by functioning in the ASH and ADL neurons that detect noxious chemicals (de Bono *et al.* 2002). We note that while OSM-9/OCR-2 are often referred to as being *required* for all ASH-mediated behaviors, in many cases the avoidance response of the presumed null mutants is reduced but not eliminated. For example, *osm-9* and *ocr-2* mutant animals retain substantial response to quinine (Hilliard *et al.* 2004; Ezak *et al.* 2010; Mehle *et al.* 2020), and even *osm-9; ocr-2* double mutants show only a partial decrement in behavioral response to bitter compounds, 1-octanol, SDS and copper (Ezak *et al.* 2010; Mehle *et al.* 2020). Underscoring the likelihood that other channels contribute to ASH-mediated responses, mechanosensory stimulation of *osm-9* and *ocr-2* mutants (alone or in combination) evoked similar electrophysiological currents as wild-type animals (Geffeney *et al.* 2011). Thus, we suggest that it would be more accurate to instead consider these channels as *contributing* to all examined ASH-mediated behaviors. The identity of the additional channel(s) that might underlie the remaining responses to chemosensory stimuli remains unknown.

Calcium imaging experiments have confirmed a role for OSM-9 and OCR-2 in ASH chemosensory signaling. The ASH calcium flux in response to 10 mM quinine is apparently eliminated in *osm-9* animals (Hilliard *et al.* 2005). Calcium signaling in response to 10 mM copper was also strongly reduced (Hilliard *et al.* 2005). Although the ASH neurons only show an ON response when presented with 1 mM CuSO_4_, a biphasic (ON and OFF) response was observed in response to higher (10  and 50 mM) concentrations when presented for extended durations (Wang *et al.* 2015). In these cases, the OFF-response was completely dependent on OSM-9, while *trpa-1* (TRPA channel mutant) animals also showed a dramatic decrease in the OFF-response (Wang *et al.* 2015). *osm-9* and *ocr-2* mutants also show a diminished ASH calcium flux in response to high pH (11.2) (Sassa *et al.* 2013), and OSM-9 contributes to both acid- and alkali-activated electrical currents in ASH (Wang *et al.* 2016).

As discussed below, OSM-9 and OCR-2 are co-expressed in the ADL sensory neurons, where they mediate the avoidance of high concentrations of the dauer pheromone component ascr#3 (Jang *et al.* 2012). Not much is known about the sensory signaling role of OSM-9/OCR-2 in the ADF neurons, but OSM-9 does contribute to PHA/PHB calcium dynamics in response to nociceptive chemosensory stimuli (Zou *et al.* 2017).

Genetic and behavioral analyses have indicated that OSM-9/OCR-2 signaling depends on and can be activated by specific polyunsaturated fatty acids (PUFAs), although the lipid-mobilizing enzyme(s) that act downstream of sensory G proteins in AWA and ASH are not yet known (Kahn-Kirby *et al.* 2004). PUFAs are required for both AWA- and ASH-mediated chemosensory behaviors, but each sensory cell may rely on different PUFAs. While the ASH neurons may use a broad set of 20-carbon PUFAs, the AWA neurons appear more selective to EPA (eicosopentanoic acid) and AA (arachidonic acid) (Kahn-Kirby *et al.* 2004). The application of exogenous PUFAs can elicit behavioral avoidance (reminiscent of ASH activation) in wild-type but not *osm-9* mutant animals, and was also sufficient to elicit ASH calcium fluxes that are dependent upon the OSM-9/OCR-2 channels (Kahn-Kirby *et al.* 2004). Together, these data suggest that the OSM-9/OCR2 TRPV channels may be directly modulated by PUFAs generated in sensory neurons in response to stimuli.

In addition to their direct role in primary signal transduction, *C. elegans* TRPV channels also regulate the transcription of sensory genes (Tobin *et al.* 2002; Zhang *et al.* 2004; Gruner *et al.* 2014). For example, *osm-9* and *ocr-2* mutant animals show decreased expression of the ODR-10 diacetyl receptor in the AWA olfactory neurons (Tobin *et al.* 2002). Although *ocr-1* single mutants show no change in *odr-10p::gfp* expression, and it is only slightly reduced in *ocr-2* animals, *ocr-1; ocr-2* double mutants show little to no *odr-10p::gfp* expression (Tobin *et al.* 2002). OCR-2 (and OSM-9, weakly) promotes *srh-234* chemoreceptor gene expression in ADL (Gruner *et al.* 2014). OSM-9 and OCR-2 are also co-expressed in the ADF, where they regulate the expression of the 5-HT biosynthetic enzyme gene *tph-1* (Zhang *et al.* 2004). While the mechanism by which these channels control gene expression is not known, it has been proposed that the TRPV channels function in activity-dependent gene expression pathways (Tobin *et al.* 2002; Zhang *et al.* 2004). In addition, OCR-2 contains a functional nuclear localization sequence in its carboxy-terminal tail (Ezak and Ferkey 2011) and OCR-2 has been proposed to regulate gene expression in ASH (Ezak *et al.* 2010).

### Voltage-gated calcium channels (VGCCs)

VGCCs are activated by membrane depolarization to mediate calcium influx. The channel is formed by a pore-forming α_1_ subunit, with its 24 transmembrane domains, that can associate with distinct auxiliary subunit combinations in different physiological contexts (Catterall 2011; Zamponi *et al.* 2015). The mammalian α_1_ subunits are classified as three types: Ca_v_1 (L-type), Ca_v_2 (non L-type; P/Q, N, and R), and Ca_v_3 (T-type) (Catterall *et al.* 2005). The *C. elegans* genome encodes one of each of these main types (Hobert 2013). EGL-19 is the sole L-type (Lee *et al.* 1997), UNC-2 is a P/Q-type (Schafer and Kenyon 1995), and CCA-1 is the only T-type (Shtonda and Avery 2005; Steger *et al.* 2005). *C. elegans* also has two distantly related α_1_ subunits, NCA-1 and NCA-2 (α_1_ U-type), as well as two α_2_δ (UNC-36, TAG-180) and two β auxiliary subunits (CCB-1 and CCB-2) (Hobert 2013). To date, only mutations in *egl-19* and *unc-2* have been shown to affect *C. elegans* chemosensory signaling.

In chemosensory neurons, EGL-19 is thought to act downstream of stimulus-evoked depolarization mediated by CNG or TRPV channels, and upstream of calcium release from internal stores (Hilliard *et al.* 2005; Kato *et al.* 2014; Larsch *et al.* 2015; Zahratka *et al.* 2015; Tanimoto *et al.* 2017). UNC-2 may act downstream of the TAX-2/TAX-4 CNG channels in the AWC olfactory neurons (Hirotsu *et al.* 2000), and also stimulates *tph-1* expression in ADF (Estevez *et al.* 2004). For additional perspective, we refer the reader to articles that model calcium signaling in *C. elegans* sensory neurons (Kuramochi and Iwasaki 2010; Mirzakhalili *et al.* 2018; Nicoletti *et al.* 2019).

In the AWA olfactory neurons, diacetyl-induced calcium responses are strongly reduced in *egl-19* (reduction-of-function) mutant animals (Larsch *et al.* 2015). In addition, recent work has revealed for the first time that AWA can fire calcium-mediated action potentials, and that these are initiated by EGL-19 (Liu *et al.* 2018a). The action potentials are likely terminated by Shaker-type potassium channels encoded by *shk-1*, together with calcium-dependent processes such as calcium channel inactivation (Liu *et al.* 2018a).

VGCCs also function in *C. elegans* nociceptors. In response to 1-octanol, the ASH somal calcium flux of *egl-19* mutant animals is strongly reduced, but unaffected in *unc-2* mutants (Zahratka *et al.* 2015). However, both EGL-19 and UNC-2 are important for octanol-induced calcium signaling in ASH axons (Zahratka *et al.* 2015). EGL-19 is also responsible for the slow time-integral component of calcium signaling in the ASH neurons following 2-nonanone exposure (Tanimoto *et al.* 2017). In a regulatory feedback loop, calcium entry through EGL-19 may inhibit ASH excitability by activating the BK-type calcium-activated potassium channel SLO-1 (Williams *et al.* 2018). Decreased EGL-19 function also affects the AWB response to 2-nonanone, such that less calcium accumulates in the AWB cell bodies of *egl-19* mutants following odorant removal (“odor down phase”) (Tanimoto *et al.* 2017).

ASEL responds to increases in NaCl concentration with an increase in calcium (dependent on TAX-2/TAX-4 in the cilium), and the resulting cilium membrane depolarization likely opens EGL-19 channels in ASEL dendrites, which may amplify the electrical signal (Shindou *et al.* 2019). Consistent with these findings, ASEL-specific RNAi knock-down of EGL-19 decreased chemotaxis toward NaCl (Shindou *et al.* 2019). Supporting a selective role for EGL-19 in ASEL, application of the EGL-19 antagonist nemadapine-A (Kwok *et al.* 2006) blocked NaCl-induced depolarization of ASEL, but not of ASER (Shindou *et al.* 2019). ASER expresses a voltage-dependent calcium current, although the identity of the channel involved is not known (Goodman *et al.* 1998).

### Other channels

#### TMC-1

The novel family of transmembrane channel-like (TMC) proteins is conserved from worms to humans (Keresztes *et al.* 2003; Kurima *et al.* 2003). Little is known about their function in mammals, beyond their role in hearing (Kawashima *et al.* 2015; Yue *et al.* 2019). *C. elegans tmc-1* encodes the transmembrane channel-like protein 1 (TMC-1) that is expressed in several sensory neurons (ASH, ADF, ASE, ADL, AQR, PQR, URX, and PHA) (Chatzigeorgiou *et al.* 2013). TMC-1 is required for sodium and lithium cation-induced attraction behaviors (Dao *et al.* 2020). TMC-1 was also shown to be required in the ASH nociceptors to mediate avoidance of high-NaCl concentrations (Chatzigeorgiou *et al.* 2013). Consistent with behavioral studies (Hukema *et al.* 2006), high concentrations of NaCl evoke a large calcium flux in the ASH neurons of wild-type animals, but this was severely diminished in *tmc-1* mutant animals (Chatzigeorgiou *et al.* 2013). As TMC-1 selectively responds to sodium (not chloride) ions and has a high-sodium permeability, it suggests that TMC-1 may itself be an ASH nociceptive salt sensor (Chatzigeorgiou *et al.* 2013). However, these results were not repeated in subsequent studies (Wang *et al.* 2016; Dao *et al.* 2020), and it is not clear what differences in assay format might be contributing factors.

Alkaline pH activates an inward current in ASH, and this excitation occurs independently of G protein signaling (Wang *et al.* 2016). However, TMC-1 (along with a minor contribution from OSM-9) contributes to alkali-activated currents in ASH (Wang *et al.* 2016). In contrast, TMC-1 function is not required for acid sensation (Wang *et al.* 2016). Ectopic expression of TMC-1 was also able to confer alkaline sensitivity (assessed via calcium imaging and evoked current) to the normally alkaline-insensitive ASI sensory neurons (Wang *et al.* 2016). Combined, these results reveal a critical role for TMC-1 in sensing noxious alkaline environments, while behavioral responses to several other ASH-detected stimuli (nose touch, CuCl_2_, high osmolarity) are unaffected in *tmc-1* mutant animals (Chatzigeorgiou *et al.* 2013).

#### DEG/ENaC sodium channels

Degenerin/epithelial Na^+^ channels (DEG/ENaC channels) are voltage-independent Na^+^ (or Na^+^/Ca^2+^) homotrimeric channels (Jasti *et al.* 2007; Ben-Shahar 2011). The *C. elegans* DEG-1 channel (Chalfie and Wolinsky 1990), most likely acting in ASK, contributes to acid avoidance behavior along with ACD-1 in glial cells (Wang *et al.* 2008). *deg-1* and *acd-1* mutant animals are also less attracted than wild-type animals to lysine, although the site of DEG-1 and ACD-1 function in this behavior has not been determined (Wang *et al.* 2008).

## Plasticity

When chemical stimuli interact with sensory receptors, they initiate two processes: a behavioral response and adaptation to that response. Sensory adaptation is a form of plasticity that leads to a decreased response to a sensory stimulus following prolonged exposure. It allows animals to respond to new or changing stimuli in their environment while ignoring persistent signals. The change in responsiveness that takes place in peripheral cells (*e.g.*, olfactory neurons) where sensory signal transduction occurs typically results in short-term physiological changes in these cells.

Sensory adaptation occurs over at least two timescales: milliseconds and seconds to tens of minutes. The initial feedback may be considered part of the sensory response and is required for taxis to a stimulus. In its absence, the animals may not be able to discern a gradient. Thus, chemotaxis behavioral studies need to be coupled with physiological analyses of the second messengers within a sensory transduction pathway in order to understand how a given signaling molecule contributes to both initial response and subsequent adaptation.

### Regulation of G protein-coupled signaling (GPCRs, G Proteins)

In one form of sensory adaptation, desensitization, GPCR signaling is inhibited at the level of the receptors by a family of serine/threonine kinases (G protein-coupled receptor kinases, GRKs) that specifically recognize and phosphorylate the activated (agonist bound) conformation of receptors. Arrestin proteins then recognize and bind to the phosphorylated receptor, “uncouple” it from G proteins, and block its reactivation. Arrestin binding can also target the activated receptor for internalization and recycling back to the cell membrane (re-sensitization). Desensitization of GPCRs by GRKs and arrestin proteins is an important means of protecting against receptor overstimulation, and it allows cells to integrate information from multiple signaling inputs and to respond to new stimuli (Pitcher *et al.* 1998; Bunemann and Hosey 1999; Ferguson 2001; Pierce and Lefkowitz 2001; Komolov and Benovic 2018).

The *C. elegans* genome encodes two GRKs (GRK-1 and GRK-2) and one arrestin (ARR-1) (Bargmann 1998; Fukuto *et al.* 2004; Palmitessa *et al.* 2005). For a review of the varied roles of the *C. elegans* GRKs, see (Wood and Ferkey 2016). To date, a role for *C. elegans* GRK-1 in regulating chemosensory GPCRs has not been identified. However, loss of GRK-2 broadly disrupts chemoattraction and chemical avoidance in *C. elegans* (Fukuto *et al.* 2004; Ezak *et al.* 2010). ASH quinine-evoked calcium fluxes are also absent in *grk-2* mutants (Fukuto *et al.* 2004). Overall, the *grk-2* phenotype (chemosensory defective) is contrary to what would be expected for loss of a negative regulator of signaling (*e.g.*, hypersensitivity). While it is possible that GRK-2 plays a positive role in chemosensory signaling, it has been proposed that there may instead be a compensatory downregulation of G protein signal transduction to protect neurons from overstimulation in the absence of GRK-2 function (Fukuto *et al.* 2004). In addition, consistent with the classical function of GRKs in desensitization, *grk-2* mutants show excessive ASH-mediated avoidance of NaCl, which counterbalances ASE-mediated chemoattraction (Hukema *et al.* 2006). Furthermore, in wild-type animals GRK-2 protein levels oscillate in a circadian manner with cyclical entrainment, and GRK-2 protein levels and sensitivity to 1-octanol are inversely related (Olmedo *et al.* 2012).

In contrast to the broad chemosensory defects of *grk-2* mutants, loss of the sole *C. elegans* β-arrestin (ARR-1) leads to the more expected adaptation phenotype (Palmitessa *et al.* 2005). Loss of ARR-1 function also leads to defective gustatory plasticity, such that *arr-1* mutants do not avoid NaCl following pre-exposure as wild-type animals do (Hukema *et al.* 2006). However, the site of ARR-1 function in this process has not been determined.

Just downstream of receptor activation, RGS GTPase activating proteins can dampen Gα signaling by binding to Gα subunits and stabilizing the transition state for GTP hydrolysis, thus accelerating their intrinsic GTPase activity (Ross and Wilkie 2000; Hollinger and Hepler 2002; Willars 2006). This leads to the termination of downstream signaling by both the Gα and the Gβγ subunits as they re-associate. Emerging studies have also identified a role for RGS proteins in the modulation of GPCR and G protein signaling in synapses (Gerber *et al.* 2016).

The *C. elegans* genome encodes 21 proteins with RGS domains, including the two GRKs (Hobert 2013). Of these, 13 genes encode canonical RGS proteins most likely to directly regulate heterotrimeric G proteins in the manner described above. However, the *in vivo* role for many of the *C. elegans* RGS proteins remains unknown, likely due to extensive functional redundancy and/or subtle roles in regulating signaling. For an extensive review of *C. elegans* RGS proteins, please see Porter and Koelle (2009).

The expression pattern of RGS-3 was key to uncovering its subtle chemosensory phenotype (Ferkey *et al.* 2007). RGS-3 is expressed in a subset of sensory neurons (ASH, ADL, AWB, AWC, ASI, ASJ, ASK, PHA, and PHB). *rgs-3* mutant animals are defective in their response to strong ASH- and AWC-detected chemosensory stimuli, but respond normally when their concentrations are decreased (Ferkey *et al.* 2007). Interestingly, the defective behavioral responses of *rgs-3* animals to ASH-detected stimuli likely result from aberrantly elevated ODR-3 and/or GPA-3 activity and increased calcium levels that lead to decreased synaptic transmission (Ferkey *et al.* 2007). However, as changes in feeding status and biogenic amine levels modulate signaling levels and sensory response, after a slightly extended time off food (30 minutes) signaling was brought into the range where the hypersensitivity of *rgs-3* mutant animals could be seen (as would be expected for loss of a negative regulator of signaling) (Krzyzanowski *et al.* 2013). *rgs-2* mutants are also hypersensitive to dilute quinine at this time-point, and ASH-selective knock-down of either *rgs-3* or *rgs-2* leads to quinine hypersensitivity (Krzyzanowski *et al.* 2013). Consistent with a role in dampening G protein signaling, overexpression of either in ASH was sufficient to decrease behavioral response to quinine (Krzyzanowski *et al.* 2013).

Animals lacking function of the RGS protein EGL-10 are also defective in their response to ASH-detected chemosensory stimuli (including copper, quinine and 1-octanol), but in this case EGL-10 acts downstream of the TRPV channel OSM-9, perhaps by modulating ASH synaptic transmission (Esposito *et al.* 2010). The avoidance defects of *egl-10* animals are suppressed by mutation of the RGS-encoding gene *eat-16*, suggesting that the two RGS proteins act in antagonistic modulatory pathways to regulate ASH sensitivity (Esposito *et al.* 2010). Similarly, the two may oppose each other in olfactory adaptation (Matsuki *et al.* 2006).

### cGMP-dependent protein kinases (PKGs)

PKGs are serine/threonine kinases that are activated by cGMP binding (Lincoln *et al.* 2001; Hofmann 2005). The *C. elegans* genome encodes two PKGs, EGL-4/PKG-1, and PKG-2 (L'Etoile *et al.* 2002; Manning 2005). These kinases have two tandem cGMP binding domains that block access to the kinase domain in the absence of cGMP binding. cGMP binding releases this inhibition, allowing phosphorylation of target protein (Kim *et al.* 2016). EGL-4 is widely expressed throughout the animal (Fujiwara *et al.* 2002) and plays varied roles in several different sensory neurons (see below). PKG-2 may have minor roles in the animal's sensory physiology (Manning 2005), but these will not be discussed further.

### EGL-4/cGMP in ASEL/R

EGL-4 may act as a regulator of the primary sensory response in the ASEL and ASER salt sensing neurons (Suzuki *et al.* 2008). As discussed above (see ASE), ASEL and ASER are ON and OFF neurons whose sensory responses are mediated by ligand binding and removal, respectively (Suzuki *et al.* 2008; Murayama *et al.* 2013; Smith *et al.* 2013; Woldemariam *et al.* 2019). Suzuki *et al.* (2008) found there is no calcium influx in either ASEL or ASER in *egl-4(n479)* mutants in response to salt upsteps or downsteps. Why EGL-4 might be required in both ASEL and ASER to open the tetrameric CNG channels comprised of TAX-2, TAX-4, and CNG-4/CHE-6 (Smith *et al.* 2013), that presumably could be opened directly by the increase in cGMP produced by guanylyl cyclase activation, is unclear. It is known from CNG channel expression studies in mammalian tissue culture that the subunits that make up the heterotetrameric channel dictate the channel's affinity for cGMP and its open probability once bound (Komatsu *et al.* 1999; Matulef and Zagotta 2003; O'Halloran *et al.* 2017). Thus, the heterotetramer that includes CNG-4/CHE-6 might require PKG phosphorylation to modulate its cGMP affinity and subsequent opening. Alternatively, or in addition to directly increasing CNG channel open probability via phosphorylation, EGL-4 may promote ASE signaling either by potentiating other parts of the signal transduction cascade to augment signaling, or by inhibiting an adaptive (negative feedback) response. EGL-4 could also act as a negative regulator of signaling such that excessive calcium signaling in *egl-4* mutant animals could stimulate a calcium-dependent negative feedback loop that ultimately inhibits ASE signaling. Such a regulatory feedback mechanism would be reminiscent of loss-of-function mutations in *grk-2* (Fukuto *et al.* 2004) and *rgs-3* (Ferkey *et al.* 2007) (see above).

### cGMP/EGL-4 in AWC

Sensory signals adapt over multiple timescales. Adaptation on the order of seconds allows the sensory neuron to respond to small increases in stimulus concentrations so that animals can climb a gradient. Failure of adaptation at this timescale partially mimics the behavioral defects of sensory signaling mutants. Sensory neurons also adapt to longer odor exposures. Wild-type animals will switch their behavioral response from being attracted to AWC-sensed odors to ignoring them if they are exposed to the odors for longer than 30 minutes in the absence of food (Bargmann *et al.* 1993; Colbert and Bargmann 1995). The period of decreased responsiveness scales with the length of odor exposure, such that odor adaptation is quickly reversible if odor exposures are for less than 60 minutes, but it becomes long lasting (hours) if the exposure lasts more than 60–80 minutes (L'Etoile *et al.* 2002; Lee *et al.* 2010). The change from attraction to indifference requires EGL-4 at each time scale, beginning as early as tens of seconds, and extending through minutes and hours (L'Etoile *et al.* 2002; Lee *et al.* 2010; Juang *et al.* 2013; O'Halloran *et al.* 2017; Levy and Bargmann 2020). The hours-long decrease in responsiveness may represent associative conditioning, as it requires pairing with starvation and is blocked by food (Torayama *et al.* 2007; Kauffman *et al.* 2011; Cho *et al.* 2016).

At very short time scales (on the order of seconds), loss of *egl-4* and failure to adapt actually leads to the inhibition of chemotaxis to butanone (Daniels *et al.* 2000; L'Etoile *et al.* 2002). In *egl-4* loss-of-function mutants, AWC exhibits increased calcium influx in response to changes in butanone concentration, relative to wild-type animals (Levy and Bargmann 2020). Conversely, *egl-4* gain-of-function mutants show reduced calcium influx (Levy and Bargmann 2020). Thus, EGL-4 sets the threshold for calcium responsiveness in the timeframe needed for odor sensation and adaptation in a gradient (Levy and Bargmann 2020).

At slightly longer time scales (tens of minutes), it is likely that EGL-4 phosphorylates cytoplasmic targets. Consistent with this possibility, the EGL-4 consensus site on TAX-2 is required for odor adaptation (L'Etoile *et al.* 2002). After exposures of longer than 60–80 minutes, EGL-4 enters the AWC nucleus (O'Halloran *et al.* 2009; Lee *et al.* 2010; Cho *et al.* 2016), and this translocation requires cGMP binding to EGL-4 and G-protein signaling (O'Halloran *et al.* 2009; Lee *et al.* 2010). However, aberrantly high levels of cGMP (due to loss of phosphodiesterases or application of membrane permeable cGMP) blocks nuclear translocation of EGL-4, even when animals are exposed to odor (O'Halloran *et al.* 2012). ODR-1 function and cilia integrity are required to keep EGL-4 in the cytoplasm until worms are exposed to odor (O'Halloran *et al.* 2012). The residues of EGL-4, the subcellular distribution of cGMP and the cofactors that regulate EGL-4's localization within the cell remain to be fully elucidated.

Once in the nucleus, EGL-4 phosphorylates the heterochromatin binding factor HPL-2 in a small RNA- and nuclear RNAi-dependent fashion (Juang *et al.* 2013). One transcriptional target downregulated by HPL-2 binding is the *odr-1* guanylyl cyclase-encoding gene (Juang *et al.* 2013). Another target, *saeg-2*, was predicted from gene expression studies using a constitutively active EGL-4 allele (Hao *et al.* 2011). SAEG-2 was recently shown to be downregulated by inherited small endogenous RNA species (Posner *et al.* 2019). Thus, in addition to phosphorylating cytoplasmic targets to modulate signaling within seconds to tens of minutes, EGL-4 may also modify gene expression in a heritable manner to affect AWC-mediated behaviors across generations.

### cGMP/EGL-4 in ASH

EGL-4 negatively regulates response of *C. elegans* to select nociceptive stimuli (Krzyzanowski *et al.* 2013). In the ASH neurons, cGMP binding to EGL-4 likely stimulates it to phosphorylate and activate RGS-2 and RGS-3, which in turn downregulate Gα (ODR-3 and/or GPA-3) signaling (Krzyzanowski *et al.* 2013). Thus, similar to loss of RGS-2 or RGS-3 function (at longer time points off food), *egl-4* mutant animals respond better than wild-type animals to several ASH-detected chemical stimuli (including quinine and 1-octanol) (Krzyzanowski *et al.* 2013). Surprisingly, the source of cGMP in this modulatory pathway appears to be other sensory neurons that are indirectly connected to ASH via a gap junction neuronal circuit (Krzyzanowski *et al.* 2016). Thus, diverse sets of environmental information may be integrated, via cGMP generation and movement through a neural gap junction network, to regulate nociceptive sensitivity.

### OSM-9 in AWC

Beyond its role in primary signal transduction in other sensory neurons, the TRPV channel OSM-9 is also required for adaptation to the AWC-sensed odorants butanone and isoamyl alcohol (Colbert and Bargmann 1995). It is still unclear how OSM-9 promotes adaptation to AWC-sensed odorants, but it acts in both cGMP-mediated and calcium-mediated plasticity pathways. First, OSM-9 acts downstream of nuclear EGL-4; adding an additional nuclear localization signal onto EGL-4 is sufficient to drive odor adaptation and causes animals to ignore all AWC-sensed odors, unless *osm-9* is also mutated (Lee *et al.* 2010). Second, downstream of calcium signaling, reduced TAX-6 calcineurin function results in failure to respond to isoamyl alcohol due to AWC being constitutively adapted; chemotaxis is restored to *tax-6* mutants upon loss of *osm-9* (Kuhara *et al.* 2002).

### Ras/MAPK (mitogen-activated protein kinase) pathway

The levels of Ras/MAPK pathway activity are important for *C. elegans* olfactory responses. For example, the most loss-of-function mutations in genes of the Ras/MAPK pathway result in mild-chemotaxis defects to diacetyl (AWA), isoamyl alcohol (AWC) and 2,4,5-trimethylthiazole (AWA and AWC) (Hirotsu *et al.* 2000). Loss of RGEF-1b function, a putative RasGRP (activating Ras GTP exchange factor), also disrupted chemotaxis to AWA- and AWC-sensed odorants (Chen *et al.* 2011). Conversely, loss of the RasGAPs GAP-1 and GAP-3 (presumed negative regulators of Ras activity) also leads to mild chemotaxis defects (Gyurkó *et al.* 2015). GAP proteins also act, in part, through LET-60 Ras to regulate learning and memory in *C. elegans* (Gyurkó *et al.* 2015), although their site of action in this context is not known.

Recruitment of the Ras/MAPK pathway downstream of primary sensory signaling can modulate behavioral sensitivity. Application of isoamyl alcohol led to activation of Ras itself within seconds, dependent upon the function of the upstream olfactory signaling components ODR-3 and TAX-2 (Uozumi *et al.* 2012). Within 10 seconds of isoamyl alcohol addition, MAP kinase is activated and this is dependent upon TAX-2/TAX-4 and UNC-2 (Hirotsu *et al.* 2000). In addition, upregulation of pathway activity (via over active LET-60/Ras or loss of MPK-1) leads to strong chemosensory defects, particularly in response to isoamyl alcohol (Hirotsu *et al.* 2000; Uozumi *et al.* 2012). This suggests that activation of the Ras/MAPK pathway following odor exposure may be an additional mechanism to downregulate the AWC-mediated olfactory response, at least in part by modulating interneuron activity (Uozumi *et al.* 2012). However, the exact mechanism by which the MAPK pathway does so is unknown. A negative feedback loop also quickly inactivates Ras, and the dynamics of Ras activation may help to tune an animal’s response to changes in odor concentration during klinotaxis (Uozumi *et al.* 2012).

## Sensing and transducing pheromone signals

**Figure 5:**
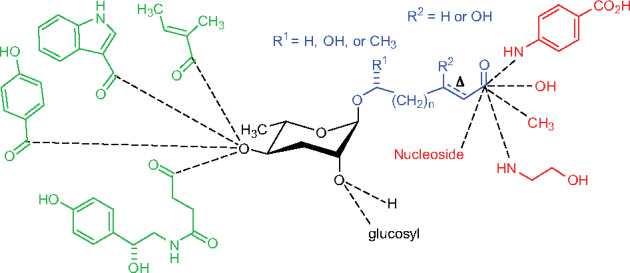
Summarized overview of the chemical structure of ascaroside pheromones. The ascarylose sugar moiety (black) is attached to a fatty acid of different chain lengths (blue). Subsets of pheromone molecules also contain additional components (indicated as R1, R2, red, and green). Reproduced from McGrath and Ruvinsky (2019).

*C. elegans* employs a remarkably rich chemical language comprised of hundreds of small molecules to communicate with conspecifics (McGrath and Ruvinsky 2019). Although the presence of molecules with biological activity in conditioned medium from *C. elegans* cultures has been known since the early 1980s (Golden and Riddle 1982, 1984c), the identities of a subset of these compounds were first described only several decades later (Jeong *et al.* 2005; Butcher *et al.* 2007; Srinivasan *et al.* 2008; Pungaliya *et al.* 2009). The major class of molecules that acts as pheromones in *C. elegans* is a structurally related family of chemicals derived from the sugar ascarylose and containing fatty acid side chains (ascarosides) (Ludewig and Schroeder 2013; Butcher 2017a; Figure 5). The specific ascarosides produced, and their relative concentrations, are regulated by the animal’s sex, developmental stage, reproductive and metabolic status, as well as experience (Butcher 2017a; Park *et al.* 2019b). Not surprisingly, individual ascarosides and ascaroside mixtures elicit diverse effects in responding animals, ranging from acute behavioral responses (“releaser effect”) to altered development and physiology via modulation of neuroendocrine signaling (“primer effect”) (Wyatt 2003). There is evidence that molecules other than ascarosides also act as pheromones, although their chemical identities have not been comprehensively elucidated (see below for further discussion and references). This section describes the current knowledge regarding the sensory neurons and molecules required for pheromone-mediated regulation of physiology and behavior. We refer the reader to several excellent recent reviews for information on pheromone biosynthesis and composition in *C. elegans* (Ludewig and Schroeder 2013; Chute and Srinivasan 2014; Butcher 2017a, b, 2019; Park *et al.* 2019b).

## Pheromone signal transduction in the regulation of development and physiology

### Regulation of dauer formation

*C. elegans* larvae develop into reproductive adults via one of two mutually exclusive developmental trajectories (Fielenbach and Antebi 2008). Under conditions of plentiful food, low temperature, and low-population density (and thus low levels of pheromone), L1 larvae progress sequentially through the subsequent L2–L4 larval stages to develop into reproductive adult hermaphrodites. However, under adverse environmental conditions, L1 larvae instead enter into the long-lived and stress-resistant dauer stage (Cassada and Russell 1975; Golden and Riddle 1982, 1984b, c). When conditions improve, dauer larvae exit the dauer stage and develop into reproductive post-dauer adults (Fielenbach and Antebi 2008). Both high concentrations of pheromone and high temperature are instructive for dauer formation (Golden and Riddle 1982, 1984a, b; Ailion and Thomas 2000), whereas food levels are generally permissive (Golden and Riddle 1984a; Neal *et al.* 2015; O'Donnell *et al.* 2018).

Of the many ascarosides that have been identified, a subset including ascr#1, ascr#2, ascr#3, ascr#5, ascr#8, and icas#9 has been shown to regulate dauer entry (Jeong *et al.* 2005; Butcher *et al.* 2007, 2008, 2009a; Srinivasan *et al.* 2008; Pungaliya *et al.* 2009). Pheromone chemoreceptors necessary for dauer formation include the GPCRs SRBC-64 and SRBC-66 expressed specifically in ASK (Kim *et al.* 2009), SRG-36 and SRG-37 expressed specifically in ASI (McGrath *et al.* 2011), and DAF-37 and DAF-38 expressed in ASI, ASK, and IL2 (Park *et al.* 2012). Although SRBC-64 and SRBC-66 act nonredundantly, SRG-36 and SRG-37 act partially redundantly to mediate dauer formation (Kim *et al.* 2009; McGrath *et al.* 2011; Lee *et al.* 2019; Table 3). DAF-37 acts in ASK to modulate dauer formation (Park *et al.* 2012), and has been suggested to heterodimerize with DAF-38 (Park *et al.* 2012; Table 3). With the exception of DAF-38 whose localization pattern has not been reported, these receptors are localized to the sensory ciliated endings of the corresponding expressing neurons (Kim *et al.* 2009; McGrath *et al.* 2011; Park *et al.* 2012).

*srbc-64* and *srbc-66* mutants exhibit strong defects in dauer formation induced by low concentrations of ascr#1, ascr#2, and ascr#3, with weaker defects in ascr#5-induced dauer entry (Kim *et al.* 2009). In contrast, *srg-36 srg-37* double mutants are specifically defective only in ascr#5-induced dauer formation (McGrath *et al.* 2011) and *daf-37* mutants exhibit specific defects in ascr#2-induced dauer entry (Park *et al.* 2012). Moreover, photoaffinity-labeled ascr#2 has been shown to bind DAF-37 expressed heterologously in mammalian cells (Park *et al.* 2012). As the conditions used to induce dauer formation greatly influence the rate of dauer entry (Golden and Riddle 1984a), it is possible that the extent of contribution of each GPCR is distinct in different conditions (Lee *et al.* 2019), in part accounting for the presence of multiple receptors for each ascaroside in the regulation of dauer formation. The requirement for receptors is also likely to be distinct at different ascaroside concentrations. The receptive range and tuning breadth of these receptors remain to be comprehensively assessed either genetically or biochemically.

**Table 3:** Summary of discussed sensory neurons and signaling molecules that transduce ascaroside signals in multiple contexts

Sensory neuron(s)	Chemoreceptors	Other signaling molecules	Context
ASK	SRG-64, SRG-66, DAF-37, DAF-38	GPA-2, GPA-3, DAF-11 TAX-2, TAX-4	Dauer formation
	DAF-37	?	Lifespan
	?	TAX-4	Attraction
ASI	SRG-36, SRG-37	GPA-2, GPA-3 DAF-11 TAX-2, TAX-4	Dauer formation
	SRX-43		Foraging
ADL	?	GPA-3	Lipid metabolism
	?	OSM-9, OCR-2	Reproductive physiology
	?	OSM-9, OCR-2	Avoidance
	?	?	Pathogen learning
ASH	TYRA-2	GPA-6	Avoidance
ASJ, ADL	SRX-44	?	Foraging
ASJ, AWB, AWC	?	TAX-2, TAX-4	Reproductive physiology

Question marks indicate neurons with a possible role in detecting a stimulus.

The signaling events downstream of the GPCRs in dauer formation are not well understood. Heterologous expression experiments suggest that ascarosides may act as inverse agonists of SRBC-64 and SRBC-66 although whether these receptors act similarly *in vivo* is unclear (Kim *et al.* 2009). The GPA-2 and GPA-3 Gα proteins have been shown to be necessary for dauer formation, and are expressed in ASK and ASI as well as in other neuron types (Zwaal *et al.* 1997; Jansen *et al.* 1999; Table 3). Mutations in the DAF-11 rGC, and TAX-2 and TAX-4 cGMP-gated channel subunits, also lead to dauer formation defects (Riddle *et al.* 1981; Vowels and Thomas 1994; Coburn and Bargmann 1996; Komatsu *et al.* 1996; Ailion and Thomas 2000; Birnby *et al.* 2000). However, since these genes are expressed broadly and are implicated in transducing dauer-regulatory chemosensory as well as thermosensory signals, it is not clear whether pheromones signal via regulation of intracellular cGMP concentrations. Interestingly, ascarosides do not appear to modulate intracellular calcium dynamics in either ASK or ASI via these receptors (Kim *et al.* 2009; McGrath *et al.* 2011). However, mis-expression of SRG-36 or SRG-37 in the ASH nociceptive neurons is sufficient to drive ascr#5-induced avoidance and regulate calcium dynamics in these neurons in adult animals (McGrath *et al.* 2011), indicating that these receptors are able to couple with calcium signaling pathways in specific contexts. Pheromone signals are integrated with food and temperature cues over hours-long timescales during development to regulate expression of neuroendocrine ligand genes such as the *daf-7* TGF-β and *daf-28* insulin-like peptide to drive the dauer decision; in this context these molecules act as primer pheromones (Ren *et al.* 1996; Schackwitz *et al.* 1996; Li *et al.* 2003; Wyatt 2003; Cornils *et al.* 2011; Schaedel *et al.* 2012; Avery 2014; Entchev *et al.* 2015; Neal *et al.* 2015; O'Donnell *et al.* 2018). How pheromone cues sensed by their cognate receptors in multiple sensory neurons are transduced, and how these signals are integrated with food and temperature information to regulate neuroendocrine signaling, remain open questions.

### Regulation of lifespan and physiology

In addition to regulating a larval developmental decision, ascarosides also regulate *C. elegans* lifespan. Exposure of late stage larvae and adult hermaphrodites to ascr#2 and ascr#3 extends lifespan and increases stress resistance; ascr#2 mediates this effect via the DAF-37 GPCR in ASK (Kawano *et al.* 2005; Ludewig *et al.* 2013; Table 3). The excreted nonascaroside molecule nacq#1 N-acetylated glutamine derivative has recently been shown to accelerate reproductive development and shorten lifespan; ascr#2 and ascr#3 antagonize these effects of nacq#1 (Ludewig *et al.* 2017, 2019; Wharam *et al.* 2017). In contrast, chemicals such as ascr#10 produced by males shorten hermaphrodite lifespan and can kill other males (Maures *et al.* 2014; Shi *et al.* 2017; Ludewig *et al.* 2019). Although ciliated sensory neurons have been implicated in sensing these small molecules in the context of lifespan regulation, the required signal transduction pathways in these neurons are largely unknown.

Exposure to ascarosides also affects additional aspects of *C. elegans* physiology. A recent study has shown that pheromone can regulate body fat stores (Hussey *et al.* 2017). In one underlying pathway, ascr#3 acts via the GPA-3 Gα protein in ADL to downregulate intracellular cAMP levels (Table 3). cAMP signaling in turn modulates cholinergic signaling to regulate expression of the *atgl-1* triglyceride lipase in intestinal cells (Hussey *et al.* 2017). Ascarosides also regulate reproductive physiology in *C. elegans* hermaphrodites. Low concentrations of ascr#3 and ascr#10 mixtures in ratios normally produced by males regulate hermaphrodite reproductive development, sperm guidance toward oocytes and aging-dependent loss of germline progenitor cells (Aprison and Ruvinsky 2015, 2016, 2017). In this context, the ascr#10 signal requires OSM-9/OCR-2 TRPV channel function in ADL in hermaphrodites; ascr#10 signaling is antagonized by ascr#3 signaling in the ASJ, AWB, and AWC neurons mediated via cGMP (Aprison and Ruvinsky 2017; Table 3). Additional ascarosides including ascr#2 and ascr#3 have been reported to also affect sperm motility in the oviduct (McKnight *et al.* 2014). Together, these observations indicate that different pheromone components, singly or together, have complex effects on diverse aspects of *C. elegans* physiology.

## Pheromone signal transduction in the regulation of behavior

### Attraction and aversion

In addition to modulating development and physiology, pheromones elicit acute behavioral responses. A key characteristic of responses such as attraction and avoidance of pheromone is that they are highly state-dependent. Pheromone-elicited behaviors are modulated by sex, environmental conditions, internal state, and past experience. A subset of the sensory neurons and circuits mediating these behavioral responses in adults has been identified, although little is known about the required sensory signaling molecules.

**Figure 6:**
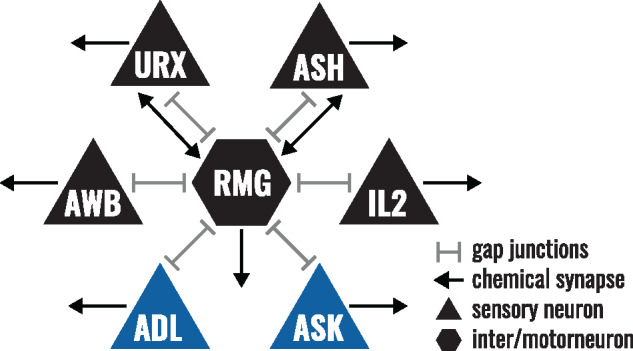
The RMG hub motor/interneurons are synaptically and electrically connected to O_2_-sensing, nociceptive and pheromone-sensing neurons. The RMG hub-and-spoke circuit integrates external and internal state information to modulate pheromone avoidance and attraction. Wiring based on White *et al.* (1986).

Both the attraction-promoting ASK and nociceptive ADL neurons respond to ascarosides (Macosko *et al.* 2009; Jang *et al.* 2012; Fenk and de Bono 2017; Wu *et al.* 2019). ASK and ADL comprise a subset of the “spokes” of a hub-and-spoke circuit motif (White *et al.* 1986; Macosko *et al.* 2009; Jang *et al.* 2012; Figure 6). In this circuit, the RMG inter/motor neuron is the central hub that is connected to spoke sensory neurons via gap junctions (Jang *et al.* 2017). In addition to being electrically coupled to RMG, each spoke sensory neuron as well as RMG itself also has chemical synapses to interneurons (White *et al.* 1986). Behavioral, genetic, and imaging analyses have indicated that sensory inputs into individual spokes of this circuit are integrated by RMG in a state-dependent manner, and via NPR-1 neuropeptide Y receptor-mediated signaling (de Bono and Bargmann 1998), to regulate both sensory responses in the spoke neurons, as well as synaptic outputs from the circuit, thereby modulating pheromone responses as a function of context (Macosko *et al.* 2009; Jang *et al.* 2012, 2017; Fenk and de Bono 2017). Interestingly, ASK also mediates attraction to the icas#3 ascaroside, but these behaviors are NPR-1- and RMG-independent (Srinivasan *et al.* 2012; Table 3).

Conditions of high NPR-1 signaling in RMG enable robust pheromone response in ADL, and weaker response in ASK, resulting in net avoidance of ascarosides, whereas upon loss of *npr-1* signaling, ASK and ADL ascaroside responses are increased and decreased, respectively, thereby driving weak attraction (Macosko *et al.* 2009; Jang *et al.* 2012; Figure 6). Pheromone responses in these sensory neurons are also further modulated by prior oxygen experience via RMG and URX, another oxygen-sensing neuronal spoke in the RMG-centered circuit (Fenk and de Bono 2017; Figure 6). Synaptic output but not sensory responses of ADL to ascr#3 are also regulated by the animal’s satiety state such that starved animals exhibit enhanced ascr#3 avoidance (Ryu *et al.* 2018). Moreover, early ascr#3 exposure has recently been shown to potentiate ascr#3 avoidance in adult hermaphrodites via modulation of ADL-driven synaptic activity (Hong *et al.* 2017). Finally, ADL but not ASK pheromone responses are sexually dimorphic (Jang *et al.* 2012). Males exhibit additional sexually dimorphic ascaroside responses that are mediated by both sex-shared and sex-specific sensory neurons (Srinivasan *et al.* 2008, 2012; Pungaliya *et al.* 2009; Narayan *et al.* 2016; Fagan *et al.* 2018). We refer the reader to the Wormbook chapter by Barr *et al.* (2018) for details on sensory neurons required for male-specific pheromone-elicted behaviors.

Transduction of pheromone signals requires the TAX-4 cGMP-gated channel and the OCR-2 and OSM-9 TRPV channels in ASK and ADL, respectively (Macosko *et al.* 2009; Jang *et al.* 2012; Table 3). The GPA-3 Gα protein has also been implicated in ascaroside avoidance behaviors (Park *et al.* 2017). Additional required signaling molecules including receptors in these sensory neurons are largely unknown, although DAF-37 has been implicated in ascr#2 sensation in ASK in adult animals (Park *et al.* 2012). Chemoreceptors mediating ascaroside responses in males have not been characterized.

Nonascaroside components also elicit avoidance and attraction in *C. elegans* (Zhou *et al.* 2017). Both male and hermaphrodite *C. elegans* avoid the internal fluid that is leaked from injured animals (Zhou *et al.* 2017). The active chemicals in this fluid are unlikely to be ascarosides but appear to be nonvolatile and require direct contact to result in repulsion (Zhou *et al.* 2017). Avoidance is mediated in part by cGMP signaling in the ASI and ASK sensory neurons but does not require the known ascaroside receptors that are expressed in these neuron types (Zhou *et al.* 2017). The presence of sperm in the hermaphrodite gonad decreases their ”sex appeal” to males via the production of nonascaroside volatile chemicals (Morsci *et al.* 2011; Leighton *et al.* 2014). Nonascaroside chemicals produced by gravid hermaphrodites also robustly attract wild-type sexually mature males at a distance but have no effect on hermaphrodite behavior (Chasnov *et al.* 2007; White *et al.* 2007). Male attraction to a subset of these ”sex pheromones” is largely mediated by the sex-shared AWA and AWC, as well as the male-specific CEM sensory neurons (Chasnov *et al.* 2007; White *et al.* 2007). Responses to volatile sex pheromones in the AWA sensory neurons have recently been shown to be mediated by the SRD-1 GPCR (Wan *et al.* 2019). SRD-1 is expressed in AWA, ASI, and ADF in adult but not larval males, with expression in AWA and ADF being sexually dimorphic (Troemel *et al.* 1995; Wan *et al.* 2019). Sex pheromone receptors in other sensory neurons in males are as yet unidentified.

### Avoidance of osas#9

A particularly intriguing class of small molecule pheromones in *C. elegans* are ascarosides that are connected to byproducts of other metabolic pathways. For instance, osas molecules are comprised of ascarosides connected to succinylated octopamine, the invertebrate analog of norepinephrine. Correlated with upregulation of octopamine by nutrient deprivation (Tao *et al.* 2016), osas#2, osas#9, and osas#10 are produced specifically by starved animals, although production appears to be restricted to L1 larvae (Artyukhin *et al.* 2013). osas#9 (derived from ascr#9) elicits robust avoidance behaviors by starved larvae and adults, suggesting that this molecule acts as a signal promoting dispersal from unfavorable conditions (Artyukhin *et al.* 2013; Chute *et al.* 2019). Interestingly, consistent with the presence of an octopamine moiety on osas#9, avoidance appears to be mediated by the TYRA-2 tyramine/octopamine receptor and the GPA-6 Gα protein in the ASH nociceptive neurons (Table 3), although receptors in addition to TYRA-2 expressed in other sensory neuron types may also contribute to the response (Rex *et al.* 2005; Chute *et al.* 2019). *tyra-2* expression is upregulated upon starvation (Chute *et al.* 2019), providing a plausible mechanism for enhanced osas#9 aversion by starved animals. These observations suggest that *C. elegans* has co-opted both a neurotransmitter and neurotransmitter receptor for inter-organismal signaling of environmental conditions, and raise the possibility that additional related molecules and transduction pathways communicate unique contextual cues.

### Regulation of olfactory behavioral plasticity

In addition to directly eliciting behaviors, pheromone experience can modulate responses to other chemical cues in adult *C. elegans* in part via regulation of sensory gene expression. For instance, it has long been known that exposure to an initially attractive chemical in the absence of food subsequently abolishes attraction to that chemical (Colbert and Bargmann 1995; Hirotsu and Iino 2005). The extent of this behavioral plasticity is modulated by prior ascaroside exposure (Yamada *et al.* 2010) and is abolished in *daf-22* mutants which fail to produce ascarosides (Golden and Riddle 1985; Butcher *et al.* 2009b; Yamada *et al.* 2010). Pheromone downregulates expression of the olfactory plasticity-antagonizing neuropeptide *snet-1* in pheromone-sensing neurons such as ASK and ASI to decrease attraction (Yamada *et al.* 2010). Pheromones also modulate expression of a subset of GPCRs in sensory neurons, although the behavioral consequence of this regulation is currently unclear (Peckol *et al.* 2001; Kim *et al.* 2009; Park *et al.* 2019a). At least a subset of pheromone-mediated modulation of sensory gene expression is mediated by known pheromone receptors such as SRBC-64 and SRBC-66 (Kim *et al.* 2009; Park *et al.* 2019a).

In a well-characterized learning paradigm, *C. elegans* learns to avoid odors associated with the pathogenic bacteria *P. aeruginosa* PA14 following a period of feeding on this bacterial strain and subsequent infection (“training”) (Zhang *et al.* 2005). Addition of a mixture of ascr#2, ascr#3, and ascr#5 to the training plates was found to significantly decrease pathogen avoidance behavior and increase pathogen resistance in *C. elegans* in trained animals in part via modulation of insulin signaling from sensory neurons such as AWA and ADL (Wu *et al.* 2019). The ascaroside mixture was shown to increase intracellular calcium in ADL but not AWA (Wu *et al.* 2019). While training with PA14 decreased pheromone responses in ADL, prior pheromone exposure was sufficient to abolish this suppression in trained animals (Wu *et al.* 2019). These observations indicate that *C. elegans* integrates information about social context and population density to adaptively modulate feeding decisions. The signaling pathways mediating pheromone responses in ADL in naïve and trained animals in this context has not been examined.

### Modulation of exploratory behavior

On a uniform concentration of bacterial food, *C. elegans* spontaneously switches between locomotory behavioral states referred to as roaming and dwelling (Fujiwara *et al.* 2002; Ben Arous *et al.* 2009). Roaming animals are active and explore the bacterial lawn, whereas dwelling animals exhibit slow speeds and restrict their movement to a small region by increasing the frequency of high-angle turns (Fujiwara *et al.* 2002; Ben Arous *et al.* 2009). In addition to satiety state (Fujiwara *et al.* 2002; Shtonda and Avery 2006; Ben Arous *et al.* 2009; Flavell *et al.* 2013), exposure to a subset of ascarosides including ascr#2, ascr#3, ascr#8, and icas#9 has been shown to regulate foraging behavior (Greene *et al.* 2016a). In the presence of relatively high concentrations of these chemicals, exploration is suppressed primarily via decreasing the fraction and duration of time animals spend in the roaming state. Analyses of natural variation in ascaroside-mediated regulation of foraging in multiple *C. elegans* strains identified the SRX-43 chemoreceptor in the ASI chemosensory neurons that specifically mediates icas#9-induced regulation of exploratory behavior (Greene *et al.* 2016a, b; Table 3). icas#9 regulates *daf-7* TGF-β and *daf-28* ILP expression in ASI (Greene *et al.* 2016a, b), indicating that as in dauer formation this ascaroside acts as a primer pheromone via SRX-43 to modulate exploratory behavior. icas#9- and SRX-43-mediated regulation of exploration is further modulated by icas#9 acting via the SRX-44 GPCR in ASJ and ADL (Greene *et al.* 2016b; Table 3).

Why might ascarosides suppress foraging? An interesting possibility has recently been proposed in the context of ascr#10-mediated suppression of exploration. The male-specific ascr#10 reduces exploratory behavior in hermaphrodites in part via increased serotonergic signaling (Aprison and Ruvinsky 2019a). Reduced roaming enhances mating success (Aprison and Ruvinsky 2019a), suggesting a plausible physiological relevance for the ascaroside-mediated suppression of foraging. Providing an intriguing example of how pheromones can additionally coordinate physiology and behavior, ascr#10-induced upregulation of serotonergic signaling also promotes egg-laying which in turn is permissive for ascr#10-mediated suppression of foraging (Aprison and Ruvinsky 2019b). Although ascr#10 has been shown to act via ADL and the OSM-9 TRPV channel to modulate reproductive physiology (Aprison and Ruvinsky 2017), whether this pathway is also involved in modulation of foraging behavior has not been established.

## Conclusions

Chemical stimuli, including odorants and tastants, provide information about individuals of the same species, food availability, food quality, and environmental threats. From the studies described here, unifying themes have emerged, as well as new questions. For example, while each *C. elegans* sensory neuron expresses multiple GPCRs, it is not yet clear how a neuron might discriminate between stimuli that couple to multiple G proteins and activate shared downstream signal transduction components. Continued receptor de-orphanization efforts may also shed light on whether *C. elegans* chemosensory GPCRs are dedicated to select chemicals, or can respond (alone or in combination) to a range of stimuli. We can now watch, in real time, how both calcium and cyclic nucleotide messages traverse the sensory neuron, which will allow analysis of the role subcellular localization of these signals may play in sensory transmission, integration (or partitioning), and regulation. Whether the dynamics of nuclear translocation of signaling and transcription factors play a role in shaping the output signals from sensory neurons is another area left to explore. Furthermore, how narrow or broad is the role for left/right neuronal asymmetry? And, while the sensory neuron itself is the site of extensive plasticity, how are signals from multiple sensory neurons integrated within the nervous system to sharpen context- and experience-dependent behavioral responses? With the continued development of tools for single-cell and circuit-level analyses, as well as computational approaches to analyze complex data sets, future work will continue the quest begun five decades ago to understand sensory signaling and behavior in the “simple” model organism, *C. elegans*.

## Appendix

### Behavioral strategies used to track toward or avoid chemical cues

*C. elegans* navigate, exploit and survive shifting environments by responding to volatile and dissolved chemical cues that may be encountered as nonuniform concentration gradients. Moreover, these signals can travel in waves or plumes from distant sources. Thus, the sensory neurons that detect these stimuli need to respond to stimuli that vary over orders of magnitude. To accurately locate the source of an attractive stimulus, the sensory neuron must also be able to adapt to the local concentration to enable response to further concentration changes and, thus, allow the animal to ascend the stimulus gradient. In theory, these neurons may also be able to integrate stimulus concentration over time in order to track discontinuous stimuli (*e.g.*, waves of odor) (Kato *et al.* 2014; Levy and Bargmann 2020).

Worms use multiple motor strategies to locate the source of attractive chemical cues and to avoid noxious ones. Location of an attractive source appears to be primarily mediated via klinotaxis and klinokinesis on a gradient. When removed from food, animals initially exhibit a brief period of area-restricted search characterized by frequent high-angle turns (Wakabayashi *et al.* 2004; Gray *et al.* 2005), which is followed by increased periods of roaming (Tsalik and Hobert 2003; Hills *et al.* 2004; Wakabayashi *et al.* 2004; Gray *et al.* 2005; Roberts *et al.* 2016). Klinotaxing animals run smoothly up a chemical gradient, gradually reorienting by biasing head swings such that they angle up the gradient (Figure A1) (Iino and Yoshida 2009; Izquierdo *et al.* 2015). This weathervane-like steering depends on chemosensory signals that modulate ongoing sinusoidal head sweeps (Iino and Yoshida 2009; Kato *et al.* 2014; Satoh *et al.* 2014; Izquierdo *et al.* 2015). Although animals maintain a constant crawling speed of 0.12 mm/s as they run directly up a salt gradient (Iino and Yoshida 2009), their speed in an olfactory gradient can be altered by odor concentration (Albrecht and Bargmann 2011).

The major contributor to chemotaxis, klinokinesis, is also termed a biased random walk and pirouette strategy (Pierce-Shimomura *et al.* 1999, 2005; Wakabayashi *et al.* 2004; Gray *et al.* 2005; Luo *et al.* 2014) (Figure A1). A pirouette is a bout of one or more coupled reversals and omega turns, during which an animal bends head to tail into the shape of the Greek letter Ω (Croll 1975b; Pierce-Shimomura *et al.* 1999; Broekmans *et al.* 2016). In general, when moving up the gradient of an attractive chemical, animals suppress the frequency of reversals and turns and increase forward run duration, whereas when moving down the gradient, animals increase reversal frequency and terminate runs. The converse strategy is used when avoiding a repellent (Tanimoto *et al.* 2017) or high-salt concentration (Kunitomo *et al.* 2013). When exiting the pirouette, the probability that the animal’s head will be oriented up the chemical gradient for an attractive chemical is higher than chance alone (Pierce-Shimomura *et al.* 1999). Each strategy depends upon the animal’s ability to monitor the change in chemical concentration as a function of time as the worm crawls through a spatial chemical gradient.

Combinations of klinotaxis and klinokinesis may be employed in response to all attractive odors and soluble cues (Iino and Yoshida 2009). Interestingly, feedback from motor neurons and the substrate animals are crawling on or swimming through may shape the strategy that animals use to locate an attractive chemical (Hendricks *et al.* 2012; Hendricks and Zhang 2013). Animals have also been observed to pause, perhaps to integrate stimuli over time (Roberts *et al.* 2016; Steuer Costa *et al.* 2019).

Worms also display acute avoidance responses when confronted with a noxious chemical cue. When presented at the nose, the worm halts forward locomotion and initiates backing within seconds. Such reversals are often coupled to an omega turn, enabling the animal to continue moving in a different direction (Croll 1975a, b). Animals exposed to noxious chemicals applied to their tails accelerate forward (Hilliard *et al.* 2002). In the wild, additional strategies, including three dimensional maneuvers, are likely to be employed (Bilbao *et al.* 2018).

#### rGC structure and activation

rGCs can be both activated and inactivated by extracellular ligands as well as by intracellular calcium binding factors, phosphorylation and ATP binding, and by effectors of G protein-coupled signaling (Sharma *et al.* 2016; Maruyama 2017). Extracellular ligands activate the single pass transmembrane rGCs by binding to their amino-terminal extracellular domain (ECD). Mammalian rGCs are obligate homodimers comprised of identical alpha and beta subunits whose activities are regulated by conformational changes upon ligand binding to the ECD (Sharma *et al.* 2016). The ECD is followed by a transmembrane domain and an intracellular kinase homology domain (KHD), then an ∼50 residue hinge region and a carboxy-terminal cyclase domain that is inactive until it dimerizes (Sharma *et al.* 2016; Maruyama 2017).

*C. elegans* chemosensory responses to soluble ligands are likely to be transduced by direct binding of ligand to the ECD of rGCs (Figure 4, D and E): sodium and hydroxyl ions to GCY-14 (ASEL) (Murayama *et al.* 2013), K^+^ to GCY-1 (ASER), Br^−^ and I^−^ to GCY-4 (ASER), and Cl^−^, Br^−^, and I^−^ to GCY-22 (ASER) (Smith *et al.* 2013). When each ECD was appended to a different intracellular rGC domain, and the fusion protein was mis-expressed in a nonnative cell type, appropriate ligand responsiveness (based on the ECD) was restored to animals that lacked the original respective rGC.

Although many rGCs may be activated by ligand binding, some, such as the human retinal rGC GUCY2D (Duda *et al.* 1996), have residues in their KHDs that allow dimerization in the absence of a ligand. This raises the possibility that some rGCs may be tonically dimerized and active, and ligand binding disrupts the dimerization to inactivate the cyclase. Consistent with this possibility, GCY-8 (which is normally expressed in the thermosensory AFD cells) was constitutively active when expressed in mammalian tissue culture cells and was inactivated by chloride binding to its ECD (Singhvi *et al.* 2016). The conserved D(F/H/Y)G motif in the KHD of GCY-8, as well as many other *C. elegans* rGCs, is thought to indicate whether a rGC has basal activity.

Constitutive basal rGC dimerization and activity, that is inactivated by ligand binding and restored by ligand release, could explain how stimulus application would silence (and removal trigger) calcium influx in a sensory neuron that expresses CNG channels and exhibits an OFF calcium response. Most *C. elegans* rGCs have residues in the hinge region that are consistent with the possibility that they are tonically active. By contrast, in vertebrate vision cGMP decreases are executed by GPCR-activated phosphodiesterases (Sharma and Duda 2014).

Intracellular regulators can also directly activate rGCs via mechanisms that are independent of ligand binding, but also result in moving the catalytic subunits together (for cGMP cyclization) or apart (for inhibition of this reaction) (Sharma *et al.* 2016; Maruyama 2017). One such proposed activator class is the Gα subunits released from seven transmembrane G protein-coupled receptors in response to their stimulation (Winger *et al.* 2008; Maruyama 2017). For example, ODR-1 (L'Etoile and Bargmann 2000) and GCY-12 (Fujiwara *et al.* 2015) may be primarily regulated through their ICDs. When the crystal structure of the cyclase domain of the soluble green algae guanylyl cyclase was solved and compared to that of an adenylyl cyclase, shared features indicated that the rGCs, like the adenylyl cyclases, could possibly be regulated (activated or inactivated) by G alpha binding to the hinge region (Winger *et al.* 2008). Olfactory signaling may depend on this type of intracellular regulation; ODR-1 is required for chemotaxis to all AWC-sensed odors typically tested, but removal of its ECD did not affect odor taxis (L'Etoile and Bargmann 2000). This indicates that ODR-1 may be directly regulated by GPCR signaling, possibly via a Gα (Figure 4C). Likewise, the ECD of GCY-12 was not required for its role in body size regulation (Fujiwara *et al.* 2015), suggesting that it also is likely activated by intracellular regulators that are downstream of other receptor pathways. Alternatively, ODR-1 and GCY-12 may heterodimerize with another rGC that is activated (or inactivated) by an extracellular ligand.

In the mammalian retina, rGCs are activated by calcium-dependent guanylyl cyclase activator proteins (GCAPs), which bind to the hinge region of rGCs at the conserved residues DIVGFTALSAESTPMQVV. By binding to this region, they promote association of the catalytic domains of the alpha and beta subunits into an active cyclase, but this activation is independent of ligand binding. The calcium that activates the retinal GCs is supplied by the opening of CNG cation channels in the dark, and this promotes the dark current (Sharma and Duda 2014). All *C. elegans* rGCs contain portions of this hinge sequence and may thus be activated by *C. elegans* calcium-activated GC regulators such as NCS-1 and others (Gomez *et al.* 2001; Wang *et al.* 2013).

Thus, there are at least two independent ways that *C. elegans* rGC activity can be regulated: one driven by ligand binding to or detachment from the extracellular domain, which triggers a cascade of structural changes that orient the cyclase domains into their active conformation, and the other by intracellular factors binding to the cyclase and hinge region. These two mechanisms can act on the same rGC at the same time.

#### rGC homodimers and heterodimers

Each individual mammalian rGC cyclase domain contains all the residues needed to coordinate magnesium and bind to GTP, but they nonetheless require dimerization to cyclize GTP into cGMP (Morton 2004). Although mammalian rGCs and *C. elegans* GCY-14 act as obligate homodimers, a number of *C. elegans* rGCs have been hypothesized to act as obligate heterodimers because they lack key GTP-binding and Mg^++^-coordinating residues that would only be provided by a complementary heterodimer (Morton 2004). Experimental support for this hypothesis comes from studies of the ICDs of the AWC-expressed rGCs ODR-1 and DAF-11, which are both required for chemotaxis to all AWC-sensed odors (Birnby *et al.* 2000; L'Etoile and Bargmann 2000). In order to determine if the ECD of ASEL-expressed GCY-14 directly responds to alkaline pH, the ECD of GCY-14 was appended to the ICD of either ODR-1 or DAF-11 (Murayama *et al.* 2013). The alkaline seeking and calcium influx (ON response) in response to a pH increase was only restored in *gcy-14* mutants upon expression of both, but not single, fusion proteins in ASEL. This indicates that the ICDs of ODR-1 and DAF-11 can heterodimerize in ASEL, and that the ECD can direct them to increase production of cGMP in response to extracellular ligand binding. Formal proof that ODR-1 and DAF-11 heterodimerize in their native cell types awaits additional experiments.

GCY-1, GCY-4, and GCY-22 may also act as either homodimers (based on sequence analysis) or heterodimers. In support of the latter possibility, mis-expression of GCY-4 in ASI restored iodide responsiveness to animals lacking ASE, but only when co-expressed with GCY-22 (Smith *et al.* 2013). Confirmation of heterodimer formation for these rGCs awaits biochemical or tissue culture experiments. The ability to heterodimerize could provide unique cGMP output patterns—possibly allowing different kinetics and downstream regulation, as well as combinatorial ligand binding. For example, if the six rGCs known to be expressed in ASER can homo- and hetero-dimerize, this would give rise to 21 distinct receptors, for a similarly wide array of possible ligands. Such diversity may make sense for an animal that relies on chemosensory cues to navigate its world.
