# Corrigendum to: Chemosensory signal transduction in *Caenorhabditis elegans*

**DOI:** 10.1093/genetics/iyab181

**Published:** 2021-11-28

**Authors:** Denise M Ferkey, Piali Sengupta, Noelle D L’Etoile

*GENETICS*, 2021, 217(3), https://doi.org/10.1093/genetics/iyab004

In the originally published version of this paper, on page 20 the first line of data in the “Chemoreceptors” column of Table 3 has now been changed to “SRBC-64, SRBC-66”. It originally read “SRG-64, SRG-66”.

All other results and conclusions of the manuscript remain unchanged.

